# Consequences of Location-Dependent Organ of Corti Micro-Mechanics

**DOI:** 10.1371/journal.pone.0133284

**Published:** 2015-08-28

**Authors:** Yanju Liu, Sheryl M. Gracewski, Jong-Hoon Nam

**Affiliations:** 1 Department of Mechanical Engineering, University of Rochester, Rochester, NY 14627, United States of America; 2 Department of Biomedical Engineering, University of Rochester, Rochester, NY 14627, United States of America; A*STAR Bioinformatics Institute, SINGAPORE

## Abstract

The cochlea performs frequency analysis and amplification of sounds. The graded stiffness of the basilar membrane along the cochlear length underlies the frequency-location relationship of the mammalian cochlea. The somatic motility of outer hair cell is central for cochlear amplification. Despite two to three orders of magnitude change in the basilar membrane stiffness, the force capacity of the outer hair cell’s somatic motility, is nearly invariant over the cochlear length. It is puzzling how actuators with a constant force capacity can operate under such a wide stiffness range. We hypothesize that the organ of Corti sets the mechanical conditions so that the outer hair cell’s somatic motility effectively interacts with the media of traveling waves—the basilar membrane and the tectorial membrane. To test this hypothesis, a computational model of the gerbil cochlea was developed that incorporates organ of Corti structural mechanics, cochlear fluid dynamics, and hair cell electro-physiology. The model simulations showed that the micro-mechanical responses of the organ of Corti are different along the cochlear length. For example, the top surface of the organ of Corti vibrated more than the bottom surface at the basal (high frequency) location, but the amplitude ratio was reversed at the apical (low frequency) location. Unlike the basilar membrane stiffness varying by a factor of 1700 along the cochlear length, the stiffness of the organ of Corti complex felt by the outer hair cell remained between 1.5 and 0.4 times the outer hair cell stiffness. The Y-shaped structure in the organ of Corti formed by outer hair cell, Deiters cell and its phalange was the primary determinant of the elastic reactance imposed on the outer hair cells. The stiffness and geometry of the Deiters cell and its phalange affected cochlear amplification differently depending on the location.

## Introduction

The organ of Corti of the mammalian cochlea is uniquely organized with structurally significant sensory receptor cells and their supporting cells that suggest their mechanical role [1,2]. The inner and outer pillar cells form a triangular tunnel throughout the cochlear length. The Deiters cells, their phalangeal processes and the outer hair cells form a repeating structural pattern resembling the truss structure of a bridge. The organ of Corti is sandwiched between two matrices reinforced with collagen fibers, the tectorial membrane (TM) and the basilar membrane (BM). The longitudinally graded stiffness of the BM forms the physical basis for the characteristic cochlear traveling waves [3], and the tonotopy of the mammalian cochlea [4,5].

The organ of Corti complex (OCC: the organ of Corti, the TM and the BM) vibrates due to hydrodynamic pressure. The relative vibrations between OCC structures deflect the stereocilia bundle of the hair cells (hair bundle) to activate the mechano-transduction channels at the tips of the hair bundle [6]. The mechano-transduction current modulates the cells’ membrane potential. While inner hair cells’ mechano-transduction results in the activation of afferent nerve fibers attached to the cell, the mechano-transduction of the outer hair cell has a different role—to provide mechanical feedback to the OCC [7]. At least two force-generating mechanisms have been identified in the outer hair cells. The action of mechano-transduction channels can create a shear force between the TM and the top surface of the organ of Corti through the hair bundle [8,9]. Upon electrical potential change across the cell’s lateral membrane, the outer hair cell generates force along the axis of the cell length called the somatic motility [10,11]. Experimental evidence supports that the somatic motility plays a crucial role in cochlear amplification [12,13]. Some theoretical studies suggest that the somatic motility is sufficient to amplify the cochlear vibrations [14–16], while others support that both active mechanisms operate together for amplification and tuning of the cochlea [17].

For the active force of the outer hair cell to amplify the vibrations of the OCC, two basic conditions must be satisfied: the force magnitude must be large enough to overcome the impedance imposed on the actuators (outer hair cells), and be fast enough to operate at high frequencies > 50 kHz in some species. Regarding the speed of force generation, the speed of hair bundle motility, limited by the adaptation speed of transduction channels, was measured up to a few kHz [18]. The cutoff frequency due to the membrane’s RC time constant was also measured up to a few kHz [19], and that measured RC time constant was shown to be small enough to amplify OCC vibrations at high frequencies above 40 kHz [20,21]. Regarding the force magnitude, recent studies pointed out the consequence of the mechanical impedance imposed on the outer hair cells for cochlear amplification [22,23]. The OCC constrains the outer hair cells and its stiffness varies at least several hundred times along the length of the cochlea [4,5,24]. Meanwhile, there are experimental data to estimate the capacity of the outer hair cell’s somatic force. The capacity of outer hair cell membrane’s force generation is determined by the areal density of prestin molecules [25,26], a transmembrane protein identified as the source of the motility [12]. It was reported that the prestin density does not vary along the cochlear length [27]. Therefore, the force capacity of the outer hair cell somatic motility is about the same throughout the cochlea. It is unclear how an actuator with a constant capacity can amplify vibrations of both stiff basal location and compliant apical location.

We investigated two correlated questions: 1) Are there any qualitative differences in OCC micro-mechanics between high and low frequency locations? 2) How do the outer hair cells with a constant active force capacity contribute to cochlear amplification at different cochlear locations with widely varying mechanical stiffness? For the analysis, a continuum mechanics-based model of the OCC [21,28,29] was further developed to incorporate the fluid dynamics of the cochlea. Our model solved three physical domains simultaneously—structural, fluid and electrical domains. A fully deformable 3-D finite element model of the OCC incorporated all structurally significant micro-structures. Cochlear fluid dynamics were solved in 2-D using the finite difference method. To analyze the relative motion between the top and bottom surfaces of the OCC, instead of a single fluid-interacting surface, two fluid-interacting surfaces were incorporated representing the top and bottom surfaces of the OCC. The model incorporated hair bundle mechano-transduction [30,31] and outer hair cell electro-motility [21]. Several characteristic differences between the basal and the apical OCC responses were identified. Despite constant active force capacity of the outer hair cell and a steep stiffness gradient along the cochlear length, our results showed amplification and tuning levels comparable to physiological observations. We provide an explanation of how the cochlea resolves the mismatch between the cochlear stiffness gradient and the outer hair cell force capacity.

## Methods

### Fluid-structure interaction model of the cochlea

**Fig 1:**
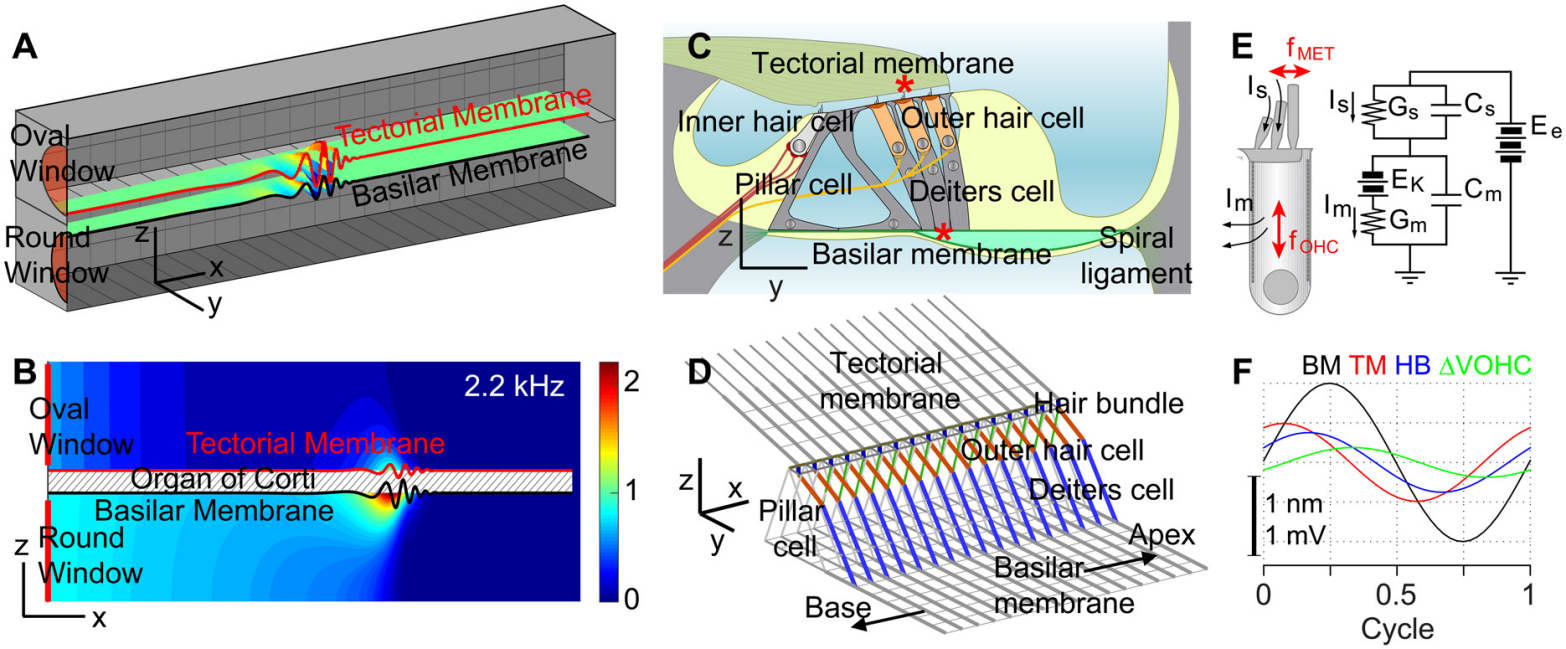
Multi-scale model of cochlear mechano-transduction. Three different physical problems are solved simultaneously: 1) cochlear fluid dynamics, 2) structural mechanics, and 3) outer hair cell electro-physiology. When the oval window was stimulated by a pure tone at 2.2 kHz, fluid-dynamical, structural, and electro-physiological responses were simulated. (**A**) The cochlear cavity was represented by a fluid-filled rectangular chamber divided into top and bottom fluid spaces by the elastic OCC. (**B**) Cochlear fluid dynamics was reduced to a 2-D domain (mesh size 10 μm in the x- and z- directions). The 2-D fluid domain interacts with the top and bottom surfaces of the 3-D OCC represented by the TM and the BM. The color contour represents pressure gain: (|*p*| - |*p*_*HT*_|)/|*p*_*OW*_|, where *p*_*HT*_ and *p*_*OW*_ are fluid pressures at the helicotrema and the oval window, respectively. (**C**) The OCC structure. Besides the BM and the TM, hair cells, pillar cells and Deiters cells are mechanically significant. (**D**) The 3-D finite element model of the OCC incorporated realistic geometrical and mechanical characteristics of the gerbil cochlea. The OCC micro-structures repeat with the longitudinal grid size of 10 μm. (**E**) Two reactive forces were incorporated with the outer hair cells—the force originating from mechano-transduction in the hair bundle (*f*_*MET*_) and the electromotility of the cell membrane (*f*_*OHC*_). (**F**) Mechanical (BM, TM and hair bundle displacements) and electrical (outer hair cell membrane potential change ΔV_OHC_) responses over a cycle.

The cochlea was represented by two rectangular chambers separated by the elastic OCC (Fig 1A). The upper chamber represents the fluid space of the scala vestibuli and the scala media, and the lower chamber represents the fluid space of the scala tympani. The stimulations were applied to the oval window in the upper chamber, and the round window in the lower chamber was considered a pressure release. At the end of the chambers opposite to the oval and round windows, the two fluid spaces were connected through an opening called the helicotrema (0.1 mm in length). The *x*-, *y*-, and *z*-axes correspond to the longitudinal, radial, and transverse direction, respectively.

While the structural domain (the OCC) was modeled in 3-D, the fluid domain was modeled in 2-D to reduce the problem size (12.1 mm long and 0.3 mm high for each fluid compartment)—omitting the y-axis of the fluid domain reduces the computational time by a factor of five. The top fluid-interacting surface was represented by the edge of the TM above the outer hair cell hair bundle, and the bottom fluid-interacting surface was represented by the centerline along the BM (asterisk markers in Fig 1C).

The OCC model incorporated 3-D structural mechanics (Fig 1D, [28]), and the electro-physiological motility of the outer hair cells (Fig 1E, [21]). Since the model details are found in the previous studies, differences and updates are summarized here. While only 1–2 mm sections of the entire cochlear coil were analyzed in the previous studies, the entire length (12 mm) of the gerbil cochlea was analyzed in this work. Structurally important components of the OCC such as the BM, the TM, the pillar cells, the Deiters cells, the reticular lamina, and the outer hair cells were represented by beam elements considering the primary direction of the structures. The hair bundles were represented by a hinged link between the apex of the outer hair cell and the TM with a rotational spring at the bundle rootlet. For simplicity, three rows of outer hair cells were merged into one row. The radial sections repeating every 10 μm were connected longitudinally by five different structures—the longitudinally running elements of the BM, the TM and the reticular lamina, and the Y-shaped structure formed by the outer hair cells and the Deiters cells. The BM edges were hinged along the inner edge and clamped along the outer edge. The edge of the TM along the spiral limbus was clamped. The apical and the basal extremities of the OCC (*x* = 0 and 12 mm) were clamped. While the boundary conditions of the BM affected the mechanical responses, changing the TM boundary condition to a hinge made little difference. The mass of the OCC was determined based on the anatomically measured cross-sectional areas of the TM, organ of Corti and BM [1]. Mechanical properties were graded along the longitudinal axis to reflect the graded geometry [1] and stiffness [4] of the OCC in the gerbil cochlea.

### Fluid structure interaction

The fluid in the top and the bottom chambers interacts with the top and the bottom surfaces of the OCC, respectively. The Navier-Stokes equations are reduced to the Laplace equation after assuming small vibrations of incompressible inviscid fluid,

$$\nabla^{\text{2}}p\text{ = 0,}$$ (1)

where ∇^2^ is the Laplace operator and *p* is the pressure. At the oval window, fluid velocity is given as input stimulation. Pressure at the round window is zero. At the fluid-structure interfaces, the fluid pressure gradient is related to the acceleration of the interacting surfaces in the z-direction (*a*_*FS*_).

$$\partial p/\partial z=-a_{32}\;\rho \;a_{FS}.$$ (2)

The factor *a*_32_ = 0.6 is to account for the interaction between the 3-D structural surfaces and 2-D fluid space after assuming a half sine wave-like deformation shape of the interacting surfaces. The mass density of the fluid (*ρ*) is considered the same as water. The fluid induced force, *f*_*Fluid*_ acting along the midline of the BM and the lateral edge of the TM (above the outer hair cell’s hair bundle) is determined by the fluid pressure such that

$$f_{Fluid}=a_{23}A_{FS}p_{FS},$$ (3)

where *p*_*FS*_ is the fluid pressure and *A*_*FS*_ is the area at the fluid-structure interface. Those two interacting points were chosen because they represent the center of the top and the bottom surfaces of the OCC. The constant *a*_23_ is a factor to convert distributed pressure to the center line, which was 0.6 (after considering the concentrated force at the center of a radial section equivalent to evenly distributed force).

### Reactive forces of the outer hair cell

The mechano-transduction channel kinetics of previous studies were linearized to obtain the relationship between the normalized transduction current ($\Delta {\hat{p}}_{o}$) and hair bundle displacement (*ξ*_*HB*_) [30,31]. According to the gating spring theory [32], the action of transduction channels results in the force (*f*_*MET*_) to deflect the hair bundle,

$$f_{MET}=f_{MET,\text{max}}\Delta {\hat{p}}_{o}\left(\xi_{HB}\right)$$ (4)

where, *f*_*MET*,*max*_ is the maximum gating force that mechano-transduction channels in a hair bundle can create collectively. The *f*_*MET*,*max*_ ranges from 100 pN at the basal end to 10 pN at the apical end [30]. The force, *f*_*MET*_, is a pair of equal and opposite forces applied at the tip and the root of hair bundle.

The membrane potential of the outer hair cell, *V*_*M*_, is modulated by $\Delta {\hat{p}}_{o}$.

$$\Delta V_{m}=\Delta {\hat{p}}_{o}G_{s}^{\text{max}}(E_{e}-V_{m}^{0})/\left(G_{s}^{\text{max}}{\hat{p}}_{o}^{0}+G_{m}^{}+s_{G}^{0}E_{K}+i\omega \;(C_{m}+C_{s}+\alpha_{0})\right),$$ (5)

where *G*_*s*_, *G*_*m*_, *C*_*s*_ and *C*_*m*_ are the conductance (*G*) and capacitance (*C*) of the transduction channel (*s*) and the lateral membrane (*m*). *E*_*e*_ and *E*_*K*_ are the endocochlear potential and the outer hair cell equilibrium potential, respectively. The voltage-dependence terms $s_{G}^{0}$ and *α*_0_ are (3.6, 0.3) nS/mV and (-2.1, 1.1) pF for *x =* (2, 10) mm, respectively. The other parameter values are presented in Table 1. See Supporting Information for further details (S1 Supporting Theory). The outer hair cell’s somatic force, *f*_*OHC*_, is proportional to the change of the membrane potential, Δ*V*_*M*_.

**Table 1:** Model parameters.

Component	Parameters	x = 2 mm	x = 10 mm	Unit	Ref.
**Basilar membrane**	Width (arcuate, pectinate)	53, 107	93, 187	μm	[69–71]
	Thickness (arcuate, pectinate)	0.6, 3	0.14, 0.7	μm	
	YM (Radial, Longitudinal)	1000, 0.4	1000, 0.1	MPa	
**Outer hair cell**	Diameter, Length	9, 20	9, 50	μm	[72,73]
	YM	0.045	0.045	MPa	
**Outer hair cell stereoclia bundle**	Height, Width	2, 8	6, 8	μm	[30,74–76]
	Stiffness	40	3	mN/m	
**Pillar cell**	Diameter	8	4	μm	[77]
	YM	10	10	MPa	
**Deiters cell**	Diameter (Body, Phalange)	10, 1.5	10, 1	μm	[77]
	YM (Body, Phalange)	0.5, 3	0.5, 3	MPa	
**Reticular lamina**	Thickness (Pillar cell, OHC)	5, 2	5, 1	μm	
	YM (Radial, Longitudinal)	10, 0.2	2, 0.05	MPa	
**Tectorial membrane**	Width (body, root)	53, 27	140, 70	μm	[70,71,78–80]
	Thickness (body, root)	30, 15	50, 25	μm	
	Radial YM (body, root)	0.2, 0.8	0.01,0.04	MPa	
	Longitudinal YM	0.002	0.002	MPa	
**Scala**	Endocochlear potential, *E*_*e*_	90	90	mV	[81]
**Stereocilia**	Max conductance, $G_{s}^{\text{max}}$	90	27	mV	[82]
	Capacitance, *C*_*S*_	2.6	9	nS	
	Open probability, $p_{o}^{0}$	0.4	0.4		
	Max reactive force, *f*_*MET*,*max*_	100	12	pN	
**Outer hair cell membrane**	Equilibrium potential, *E*_*K*_	75	75	mV	[82]
	Resting potential, $V_{m}^{0}$	-53	-37	mV	
	Conductance, *G*_*m*_	230	39	nS	
	Capacitance, *C*_*m*_	4.3	15	pF	
	Active gain, g_*OHC*_	0.1	0.1	nN/mV

$$f_{OHC}=g_{OHC}\Delta V_{M},$$ (6)

where *g*_*OHC*_ is a constant to represent the outer hair cell’s electro-mechanical gain. A constant value of *g*_*OHC*_ = 0.1 nN/mV [33] was used independent of the location. The outer hair cell’s somatic force *f*_*OHC*_ was applied along the axis of the outer hair cell as an equal and opposite force at the apical and the basal ends of the cell body so that depolarization (positive Δ*V*_*M*_) shrinks the cell.

### Combining finite element and finite difference analysis

After assuming small sinusoidal excitation at the angular frequency ω, the discretized equation of motion of the OCC is written in terms of mass (**M**), damping (**C**) and stiffness (**K**).

$$\left(K-\omega^{\text{2}}M\text{+}i\omega C\right)x\;=f_{Fluid}\text{+}f_{OHC}\text{+}f_{MET},$$ (7)

where **x** is the vector of nodal displacements. The three terms on the right hand side of Eq (7) are the force vectors due to fluid pressure, outer hair cell somatic motility, and hair bundle motility originating from transduction channel adaptations, respectively. The damping matrix **C** was approximated as **C** *= α*_*C*_ **K**, where the coefficient varies exponentially along the cochlear length. The chosen coefficients at the base and the apex were *α*_*C*_ = 1.5 μs at *x* = 2 mm, and 72 μs at *x* = 10 mm corresponding to viscous damping values of 850 nN·s/m and 330 nN·s/m per 10 μm section, respectively. Although the fluid was modeled as inviscid, this structural damping term included the effect of energy dissipation due to fluid viscosity. The *α*_*C*_ values were determined so that the quality factor of the passive OCC vibrations is comparable to experimentally measured values.

The fluid mechanics, structural mechanics and outer hair cell electro-physiology were solved simultaneously in the frequency domain. To summarize, the unknowns in the present cochlear model are: fluid pressure **p** according to the finite difference formulation of the fluid domain (Eq (1)), displacement **x** of the OCC finite element model (Eq (7)) and the electrical properties of the outer hair cell **e** comprised of the normalized transduction current and the membrane potential ($\Delta {\hat{p}}_{o}$ in Eq (4) and $\Delta V_{m}$ in Eq (6)). The combined governing equation is

$$\left[\begin{array}{ccc} A_{pp} & A_{px} & 0\\ A_{xp} & A_{xx} & A_{xe}\\ 0 & A_{ex} & A_{ee} \end{array}\right]\left[\begin{array}{c} p\\ x\\ e \end{array}\right]=\left[\begin{array}{c} b_{OW}\\ 0\\ 0 \end{array}\right].$$ (8)

Sub-matrices in Eq (8) correspond to the governing equations as follows. **A**_***pp***_ represents discretized Laplace equation of Eq (1). **A**_***px***_ represents the boundary conditions provided by the OCC fluid-interacting surfaces in Eq (2). **A**_***xp***_ represents the force boundary conditions of the OCC determined by the fluid pressure in Eq (3). **A**_***xx***_ is the coefficient matrix in the left hand side of the OCC equations of motion in Eq (7). **A**_***xe***_ is the coefficient matrix to form the reactive force of the outer hair cells described by Eqs (4) and (6). **A**_***ex***_ and **A**_***ee***_ are coefficient matrices to describe the relationship between outer hair cell’s stereociliar bundle displacements and mechano-transduction currents. The active feedback from the outer hair cell is eliminated by setting **A**_***xe***_ zero or putting *f*_*MET*,*max*_ *= g*_*OHC*_ = 0 in Eqs (4) and (6). **b**_*OW*_ is the velocity boundary condition at the oval window.

To match the grid size of the finite element model of the OCC, the fluid domain has a grid size of Δ*x* = Δ*z* = 10 μm. The combined problem has about 230×10^3^ degrees of freedom (DOF). The problem size is reduced to 160×10^3^ DOF after removing **p** in the second row of Eq (8) using the first row of the equation to obtain,

$$\left[\begin{array}{cc} A_{f}\text{+}A_{xx} & A_{xe}\\ A_{ex} & A_{ee} \end{array}\right]\left[\begin{array}{c} x\\ e \end{array}\right]=\left[\begin{array}{c} f_{f}\\ 0 \end{array}\right],$$ (9)

where **A**_*f*_ is the effective fluid reactance and **f**_*f*_ is the fluid force from the fluid domain that were obtained from $A_{f}=-A_{xp}A_{pp}^{\text{-1}}A_{px}$ and $f_{f}=-A_{xp}A_{pp}^{\text{-1}}b_{OW}$.

Model parameters are summarized in Table 1. The OCC electro-mechanical parameters were the same as the previous work [29] with some adjustments in the single channel gating force and the TM’s elastic moduli. The TM’s elastic moduli were chosen to achieve the highest quality factor of frequency tuning (Fig B in S1 Supporting Theory).

The code written in Matlab (Mathworks, Natick, MA) was run on a cluster of computer nodes (each with 2 Intel Xeon E5-2695 processors, 16 GB physical memory, BlueHive system, University of Rochester). It took about 50 minutes to assemble the sub-matrices in Eq (8) and about 4 minutes to solve for each stimulating frequency. The source code of this work can be found at a public repository*, and up-to-date version will be distributed through the corresponding author’s research webpage.

*Hyperlink: https://drive.google.com/folderview?id=0B-b6cTQy5C1NfjZEcUxETFd3cVdIU2RrYURveUUtdW1seDd4bFF4N0R0UDAxemEwT3pNSE0&usp=sharing

## Results

Results presented in this study were obtained from frequency domain analyses that predict the responses of the OCC to small perturbations. Two active electro-mechanical feedback mechanisms of the outer hair cell were incorporated: somatic electromotility and hair bundle motility. By ‘passive’, we imply the condition that the two mechanisms are deactivated, or *f*_*MET*,*max*_ = *g*_*OHC*_ = 0 in Eqs (4) and (6). Responses at three longitudinal locations: 2, 6, and 10 mm from the base are referred to as the base, middle, and apex.

### Responses to pure tone stimulations

A representative result, including fluid dynamical, structural and electrical responses to a 2.2 kHz pure tone stimulation, is shown in Fig 1. This frequency was chosen because two pressure components, the fast pressure component (instant pressure because of incompressible fluid assumption) and the pressure component due to the OCC vibrations, look comparable. To demonstrate the pressure field due to the OCC vibrations more clearly, the pressure at the helicotrema was subtracted from the fluid pressure (Fig 1B, Fig D in S1 Supporting Theory). The gradient of fluid pressure amplitude was localized to near the peak responding location. The pressure amplitude was asymmetrical with respect to the OCC because the top surface of the OCC vibrated less than the bottom surface at this frequency (the BM and the TM in Fig 1F). For the rest of this paper, we focus on micro-mechanical responses of the OCC instead of fluid pressure or the outer hair cell’s electrical responses.

**Fig 2:**
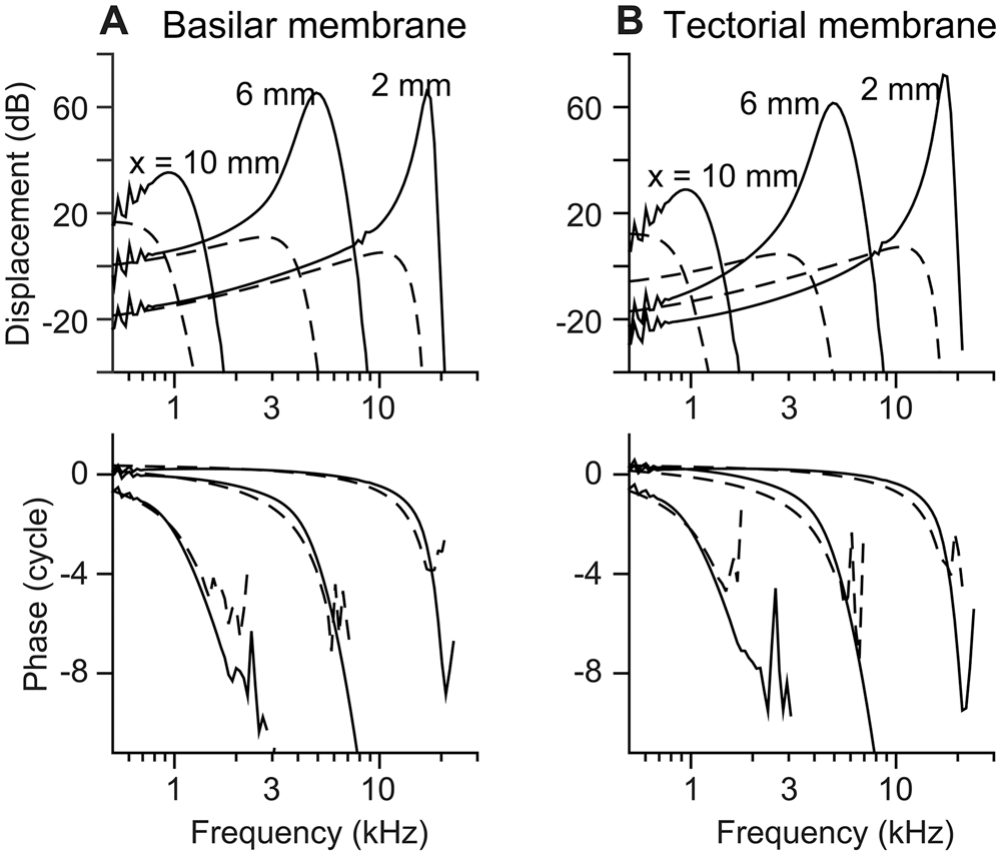
Pure tone responses of different OCC structures at three different locations. (**A**) BM transverse displacement measured at the midpoint below the Deiters cell was indicated in dB re. oval window displacement. (**B**) TM transverse displacement measured above the outer hair cell stereciliar bundle. Top panels: displacement gain in dB with respect to the stapes. Bottom panels: phase with respect to the stapes. Solid and dashed curves are active and passive responses, respectively. Three locations (x = 2, 6, and 10 mm) were chosen to represent the base, middle, and apical responses.

To demonstrate the responses of the top and bottom surfaces of the organ of Corti, the BM and TM responses were shown next to each other (Fig 2). When stimulated by pure tones at the stapes, both the BM and the TM formed traveling displacement waves indicated by more than one cycle of phase delay as the stimulating frequency increases. The damping coefficient, which affected the tuning quality, was determined so that the simulated passive responses match the measured quality factor of the cochlea under high sound pressure-level stimulations (> 80 dB) ranging from 1.5 in the base to 0.5 in the apex [34–36]. The tuning quality was higher when active—Q_10dB_ of 7.7, 3.0 and to 1.7 at *x* = 2, 6 and 10 mm, respectively. The active BM responses were greater than the passive responses by 60, 55 and 16 dB at *x* = 2, 6, and 10 mm, respectively. The TM’s amplification and tuning level were equal to or slightly greater than those of the BM. When active, the best responding frequency at *x* = 2, 6 and 10 mm was 17, 4.9 and 0.9 kHz, respectively. When passive, the best frequency shifted to 10.2, 2.7 and 0.5 kHz at *x* = 2, 6 and 10 mm, respectively. There were slight difference between the BM and the TM best frequencies (< 0.1 octave).

**Fig 3:**
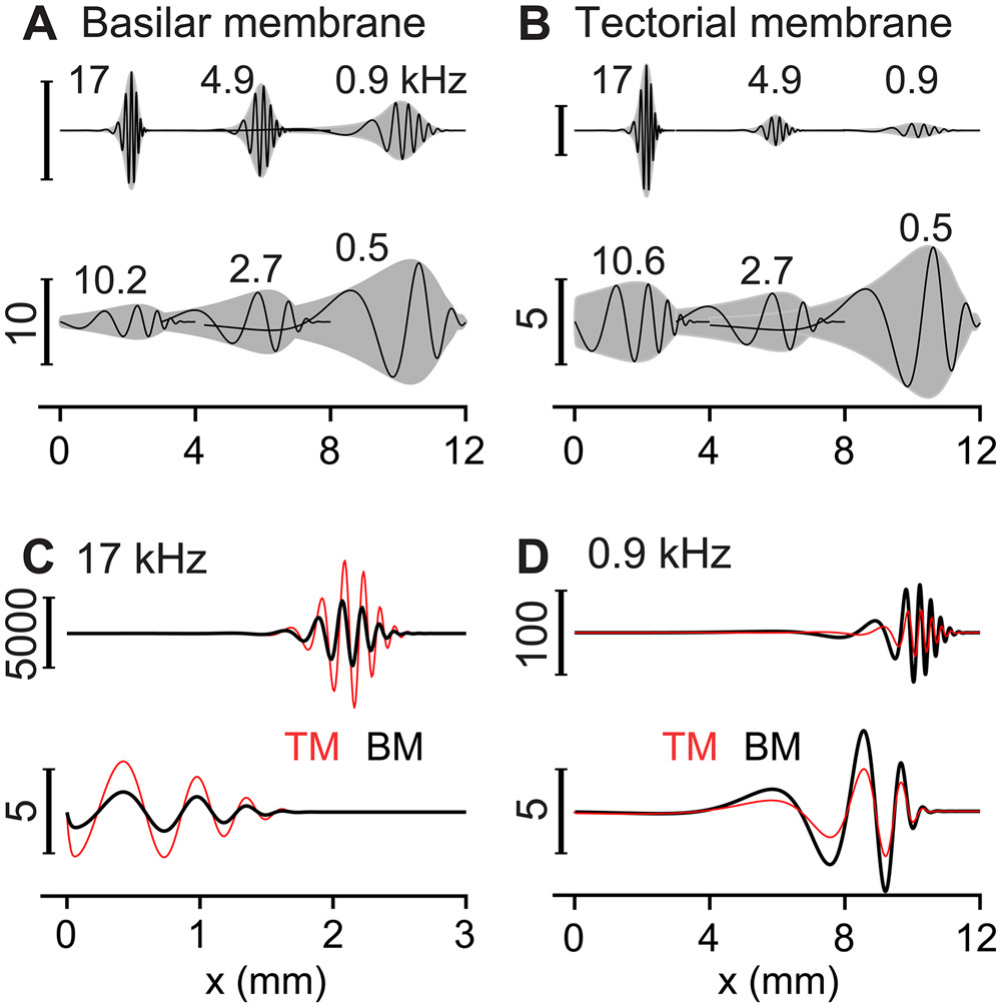
Traveling waves. (**A**) BM vibration patterns at three different stimulation frequencies when active (top) or passive (bottom). The shaded areas are the envelope of the traveling waves. Labels are the best frequencies at x = 2, 6 and 10 mm. (**B**) TM vibration patterns. (**C**) Comparison of the BM and the TM vibration patterns when stimulated at 17 kHz. (**D**) Comparison of the BM and the TM vibration patterns when stimulated at 0.9 kHz. For each panel, the top and bottom plots represent the active and passive response. The numbers next to the scale bar indicate the amplification factor (re. oval window displacement). The scale bars in the top plots of panel A and B correspond to 4000/4000/20 for the base/middle/apex curves, respectively.

The traveling waves were plotted in the spatial domain in Fig 3. At the corresponding best frequencies for the 3 locations, the wavelengths of the BM and the TM traveling waves near the peak were smaller in the basal location than the wavelength in the apex (Fig 3A and 3B). Because of the cochlear tonotopy, the characteristics of the tuning curves (along the frequency axis) in Fig 2 translate to those of the traveling waves along the spatial (*x*) axis. For example, the width (or sharpness) of the tuning curve corresponds to the width of the traveling wave envelope. The tuning curve was steeper on the high frequency side, which corresponds to the steeper traveling wave envelope on the apical side. At the same location, the best responding frequency was lower when passive than active. Likewise, at the same stimulating frequency, the peak responding location occurred more apically when active than passive. Taking advantage of the space-frequency correlation, the phase of mechanical responses was obtained along the continuous spatial domain, then transformed to the frequency axis in Fig 2 to avoid the ambiguity of tracing phase accumulation.

Two interesting trends appeared to be more pronounced in the spatial domain plots (Fig 3) than in the frequency domain plots (Fig 2). First, the TM was displaced more than the BM in the base, but it was opposite in the apex. Second, the TM traveling waves led the BM waves when the OCC was active (Fig 3C), but there was little phase difference when passive. These two characteristics related to the relative motion between the top and bottom surfaces of the OCC were further investigated.

### Relative motion between OCC structures

**Fig 4:**
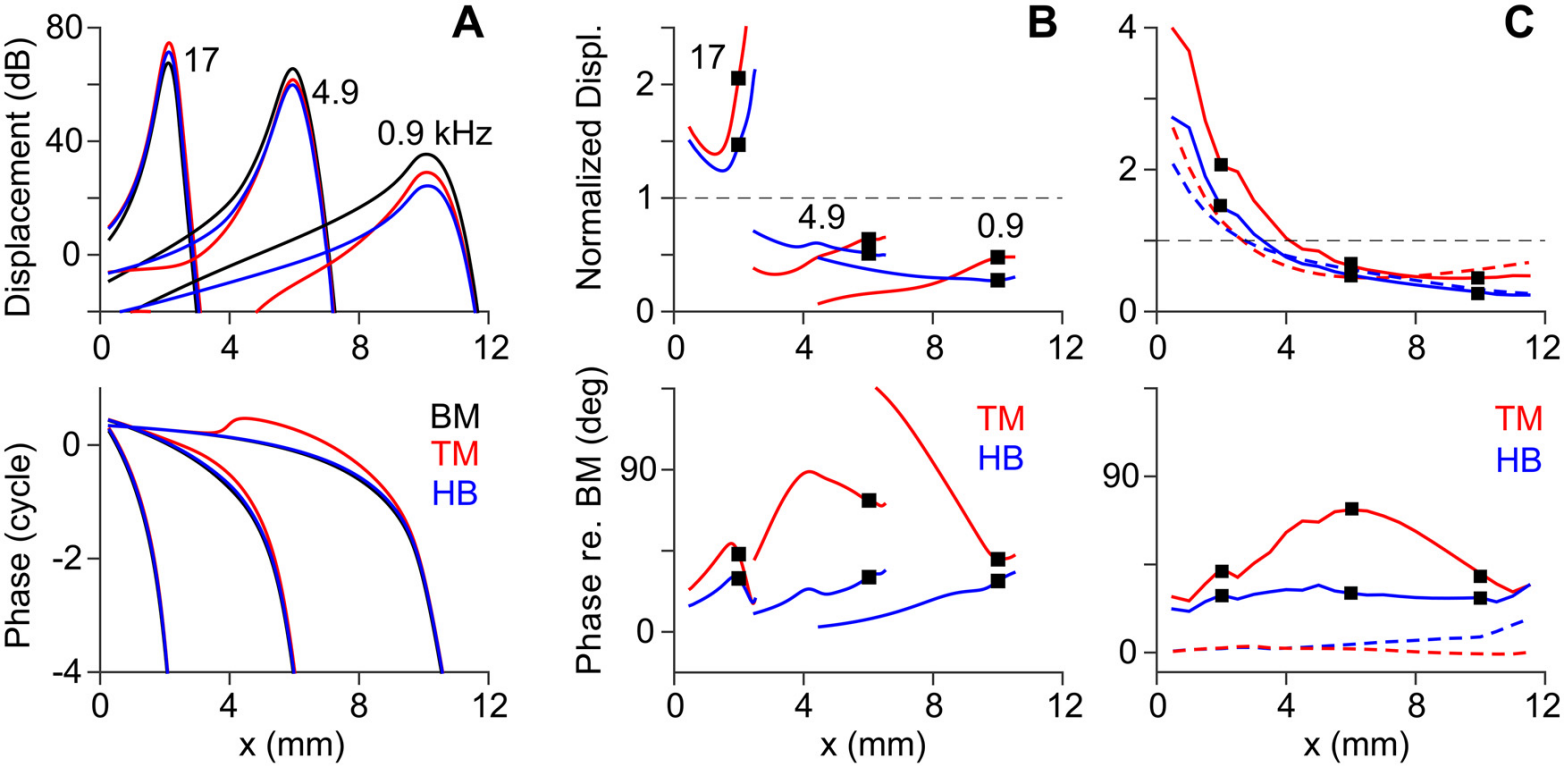
Relative motion between OCC structures. (**A**) Tone responses of the BM, TM and hair bundle (HB) at the base (17.5 kHz), middle (4.4 kHz) and apex (0.8 kHz). Top: displacement gain re. stapes. Bottom: phase re. stapes. (**B**) Responses in (A) plotted with respect to the BM—the TM and hair bundle displacement were normalized with the BM displacement; their phase with respect to the BM. (**C**) Relative responses of the TM and hair bundle re. BM at best frequencies. Broken curves are passive responses.

When passive (without outer hair cell’s motility), the micro-structures in the OCC vibrated in phase, but when active, there were out-of-phase motions between the OCC micro-structures. In Fig 4, absolute and relative responses of the OCC structures such as the TM, the hair bundle and the BM were presented (solid curves are active responses, and broken curves in Fig 4C are passive responses). The amplitudes and phases of the BM, TM, and hair bundle vibrations to stimulations at three different frequencies were compared in Fig 4A. The response curves showed similar patterns, but did not overlap exactly. To present the relative motions between OCC structures more clearly, the magnitude and phase of the TM and hair bundle motion relative to the BM motion were plotted in Fig 4B—the TM and hair bundle displacements in Fig 4A were normalized with the BM displacement, and the phase of BM displacement was subtracted from the phases of TM and hair bundle displacements. The normalized TM displacement was greater than 1 in the base, but less than 1 in the middle and apical locations. The TM and hair bundle displacements led the BM displacement. At the peak, the TM led the BM displacement by 43, 73 and 40 degrees for stimulating frequencies of 17, 4.9 and 0.9 kHz, respectively. The hair bundle displacement led the BM’s by 29, 30 and 28 degrees for those respective frequencies. The TM’s phase lead over the BM changed non-monotonically as its displacement wave propagated toward the apex so that the maximum phase lead occurred at 0.2 to 4.4 mm basal to the peak responding locations.

In Fig 4C, the normalized TM and hair bundle displacements and phases at the best frequency of each location were presented (the square markers in Fig 4B and 4C correspond to each other). In addition, the results of the passive cochlea were plotted with broken lines. Overall, the relative displacement of the TM and hair bundle decreased along the cochlear length. There was a qualitative difference between the basal and the apical responses: the TM and hair bundle vibrated more than the BM in the base, but it was opposite in the apex. This rend was stronger when active than passive (solid and broken curves, respectively, Fig 4C). The transition happened between 4 and 8 mm from the basal end. The phase plot (Fig 4C bottom) showed the most prominent difference between the active and passive responses. The structures in the passive OCC vibrated in phase while the active OCC structures vibrated with different phases. In the active OCC, the TM led the BM vibrations by 30–70 degrees, and the hair bundle led the BM vibrations by ~30 degrees.

**Fig 5:**
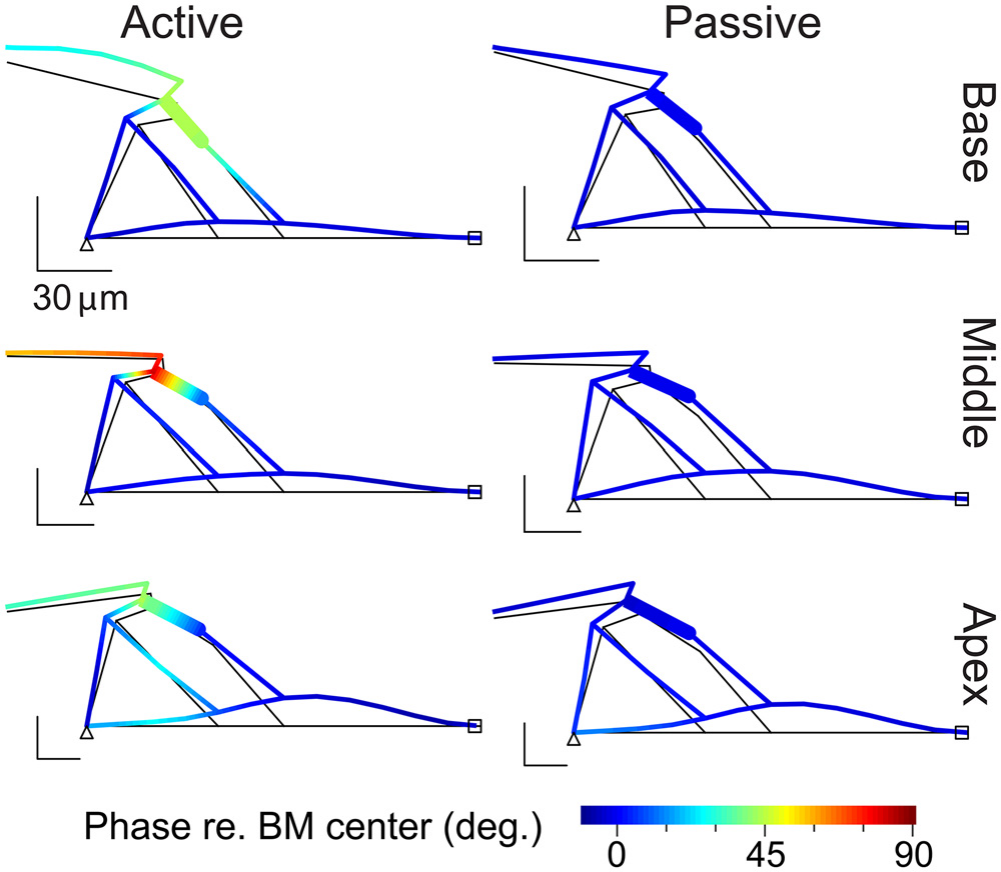
Phase difference between OCC structures. The deformed shape of the vibrating OCC is taken when the BM is displaced most under pure tone stimulation at the best frequency. Colors of the deformed OCC structures indicate the phase of the structures’ transverse (z) displacement with respect to the BM displacement below the Deiters cell. Six cases represent when the cochlea is active or passive at three different longitudinal locations corresponding to the six peak responses in Fig 2A. The thin black lines indicate the original (non-deformed) geometry. Because of the outer hair cell is tilted out of plane, the structures shown are not exactly in plane. See the movie clips.

To further demonstrate the relative motions between OCC fine structures, we plotted deformation patterns of the OCC cross-section at the best frequencies of three locations (base, middle and apex) with and without the outer hair cell’s active feedback in Fig 5 (also see S1 Movie Clip). The deformation patterns were plotted at the moment when the BM was displaced most toward the scala vestibuli. The colors of the plot correspond to the phase of the OCC structures’ transverse displacement relative to the BM displacement. When the OCC was active, there was a phase variation greater than 30 degrees within the OCC. The structural component that underwent the greatest phase change was the outer hair cell. When the OCC was passive, there was barely any phase difference such that all structures within the OCC vibrated in phase. When active hair bundle motility was turned off while the outer hair cell’s somatic motility was on (*f*_*MET*_ = 0 and *f*_*OHC*_ ≠ 0), the phase transition from the bottom to the top of the OCC persisted, but with the opposite condition (*f*_*MET*_ ≠ 0 and *f*_*OHC*_ = 0), the phase transition disappeared, similar to the passive case (right column of Fig 5). Similar responses were reported previously [21]. We conclude that the outer hair cell’s somatic motility effectively separates the vibrations of the top of the OCC from the BM vibrations. As a result, the TM leads the BM vibrations when the outer hair cell’s somatic reaction force is significant compared to the fluid pressure (or when the sound pressure level is not high enough to saturate outer hair cell mechano-transduction).

Two characteristics in the vibration modes of OCC structures were predicted in Fig 5. First, the bending deformation of the TM was observed at the base when the OCC is active, but not in the apex. Because the TM vibration modes can affect the inner hair cell’s mechano-transduction [37], this observation implies that the neural responses in the base and the apex can be different. Second, the BM vibration patterns were different depending on location. The maximum BM displacement occurred near the arcuate zone in the base, but the pectinate zone was displaced more in the apex. This BM vibration pattern was independent of outer hair cell motility. This difference is ascribed to the relative stiffness of the TM and the organ of Corti as compared to the BM. The BM stiffness at the base was dominant, but not at apical locations. This location-dependent stiffness contribution is further investigated in the following section.

### Stiffness and frequency gradient along the cochlear length

**Fig 6:**
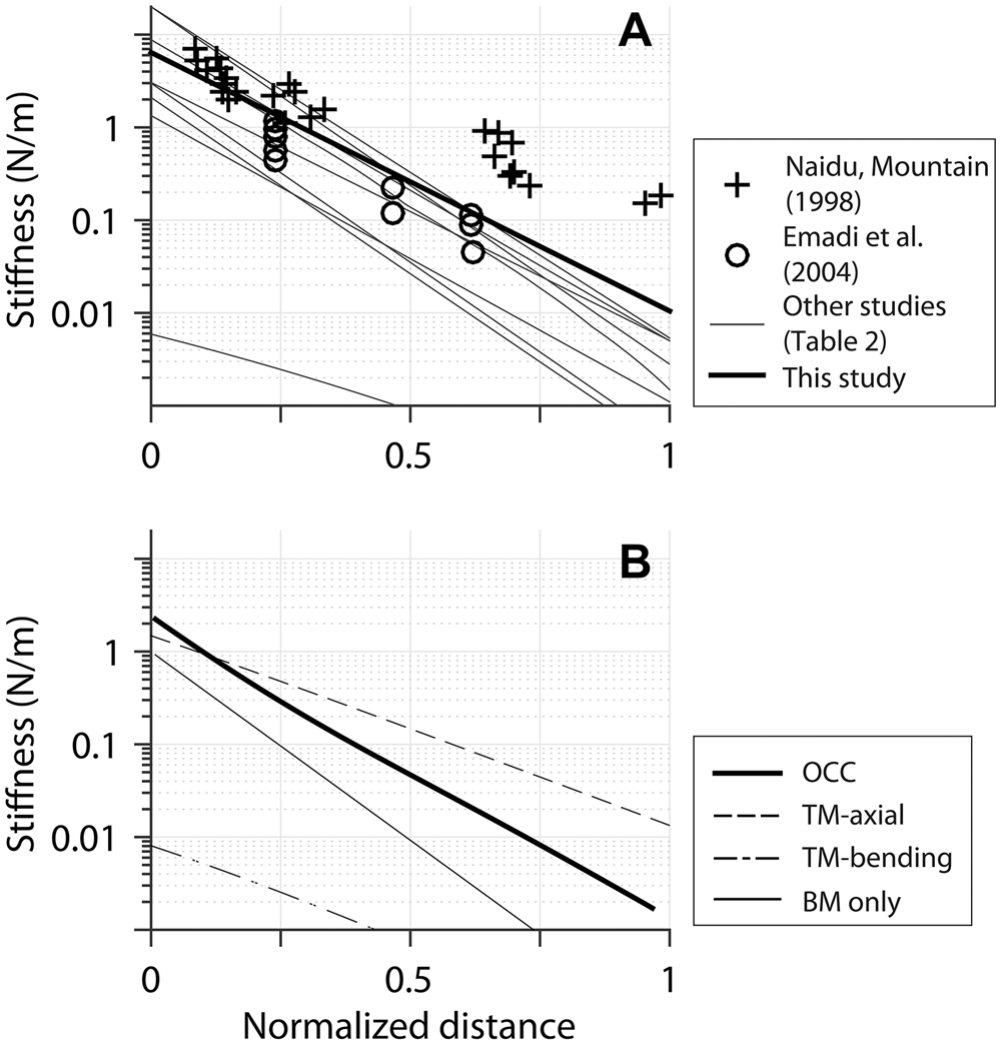
Stiffness of OCC structures along the length. (**A**) OCC stiffness versus location relations of this study, two experiments [4,24] and other theoretical studies [15,28,39,55,58,66–68]. (**B**) Stiffness of OCC structures along the cochlear length. Stiffness values per 10 μm span were presented for different structures. OCC: entire OCC. TM axial: axial stiffness of the TM. TM bending: cantilever stiffness of the TM. BM only: stiffness of the BM. kOCC_OHC: Stiffness of the OCC felt by 3 outer hair cells. kOCC_HB: Stiffness of the OCC felt by 3 hair bundles. (**C**) kOCC_OHC and kOCC_HB in (B) were normalized with the outer hair cell axial stiffness and the hair bundle stiffness, respectively.

Many existing models represented the OCC mechanics with a series of two DOF systems [38–41]. Usually, the two resonators corresponded to the BM and the TM. These two DOF models capture the primary characteristics of the cochlea such as tuning and amplification. But, there is ambiguity in defining the stiffness and mass of the mechanical model because of lacking experimental data. The stiffness properties of our finite element model were compared with those in other cochlear mechanical models and experiments (Fig 6, Table 2).

**Table 2:** Mechanical properties of the OCC in literature.

Ref.	Stiffness (*k*)				Structural mass (*m*_*S*_)				(k/m_s_)^0.5^/2π [kHz]	Species‡
	As presented *x* in [cm]	[mN/m] per 10 μm	B-A Ratio	Grad. [dB/oct]	As presented *x* in [cm]	[ng] per 10 μm	A-B Ratio	Grad. [dB/oct]		
[39]	BM: 1.1×10^9^e^-4x^	8800~5	1800	7.3	3×10^−3^	24~111	5	-1.5	96~1.1	Cat
	TM: 7×10^6^e^-4.4x^[dyn/cm^3^]	56000~13	4300	8.2	0.5×10^−3^ [g/cm^2^]	4~18.5	5	-1.5	600~4.2	
[58]	2×10^5^×10^−1.862x^ [kg/m/s^2^]	20000~5.36	3700	9.1	1.5~5[10^−6^ kg/m]	15~50	3.3	-1.0	186~0.05	Guinea pig
[66]	2×10^4^e^-3x^ [mg/(mm^2^ms^2^)]	20000~2.8	7300	11	0.5 [mg/mm^2^]	500~2500	5	-1.9	32~0.17	Human
[67]	3.0×10^9^×e^-3.0x^ [N/ m^3^] (BM values)	3000~0.41	7300	11	0.3 [kg/m^2^]	300~1500	5	-1.9	16~0.08	Human
[49]	Their Eq (13) [N/m^2^]	6500~1.5	4400	9.7	Their Eq (14)^$^	6~1.3	0.22	1.8	166~5.4	Gerbil
[83]	*k*_0_ *e* ^-3.5*x*^ [N/m^2^] ^#^	BM: 2100~3.24	648	7.2	*m*_0_ *e* ^0.5*x*^ [kg/m]^#^	2.8	1	0	138~0.5	Guinea pig
		TMs: 460~0.71				24~60	2.5	-1.0	23~0.05	
[15]	(590,40,1.6)×10^3^ [g/s^2^/cm^2^], *x* = (0,1.75,3.5)	5.9~0.08	74	5.4	(3.8,28,210) ×10^−5^ [g/cm^2^]	0.38~105	276	-7	20~0.14	Human
[28]	(1690, 25) [mN/m], *x* = (0.2,1)	1340~1.1 (412,3.66)	1200	8.2	(110,350)^&^ [ng] per 10 μm	15~178* (22.6,118)	12	-2.9	48~0.4	Gerbil
[59]	5.5e^-3.464*x*^ [N/m] per 18 μm	3050e^-3.464x^	607	7.1	*k*/(2π⋅BF)^2^	40~46200	1150	-6.3	44~0.05	Guinea Pig
[4]	(0.73,0.2,0.08) [N/m]*x* = (0.29,0.55,0.73)	1160~0.9 (144, 30.1, 11)	1290	8.3^†^	─	─	─	─	─	Gerbil
[24]	(3.2, 0.5,0.15) [N/m] *x* = (0.2,0.8,1.18)	2170~17.8 (720, 67.4, 19.0)	122	5.6^†^	─	─	─	─	─	Gerbil
This study	(2300, 31) [mN/m] *x* = (0.2, 1)	2400~1.48(565, 4.47)	1620	8.6	65.5~347^&^ [ng] per 10 μm	27.0~223*	4.0	-2.4	51~0.4	Gerbil

^#^ Coefficients for three different DoFs are *k*_*0*_ = (210, 46, 17)×10^3^, and *m*_*0*_ = (-, 2.4, 1.2)×10^−6^, the mass of the BM DoF was constant at *m*_*BM*_ = 2.8×10^−7^ [kg/m]

^†^ The stiffness reported was considered the point stiffness and had been converted into stiffness per 10 μm section for fair comparison, see text for more.

^$^ Their Eq (14) was corrected to *m = 2bρh/π* per unit length based on Lim and Steele [84].

^&^ Anatomical mass = (cross-sectional area of the OCC) × (10 μm) × (mass density).

* Effective structural mass *m*_*s*_ = *k*/(2π⋅BF) ^2^, where the BF is the best frequency of the passive OCC.

^‡^ Mass and stiffness gradient were calculated using the audible frequency range of the corresponding species. The audible frequency range is (60~0.125)/(45~0.2)/(16~0.13)/(25~0.09)/(45~0.25) in [kHz] for cat/guinea pig/human/chinchilla/gerbil, respectively

The gerbil cochlea was chosen for our study mainly because there are anatomical and mechanical data from which we could build and verify our finite element model. In particular, there are stiffness measurements along different longitudinal locations of the gerbil cochlea [4,24]. The stiffness of our OCC model, other models and the two experimental results were compared in Fig 6A. Because different models represented different species, normalized position was used for the horizontal axis. The stiffness gradient of our model matches Emadi et al.’s data [4], but the stiffness values were increased to obtain the physiological frequency range of the gerbil cochlea (0.25–45 kHz, [42]). When Naidu-Mountain’s values were used, our model could not achieve lower than 1 kHz best frequency at the apical end.

In the present model, structures repeat every 10 μm along the length. Through static analyses, the stiffnesses of OCC structures per 10 μm section were obtained and presented in Fig 6B. Because our OCC model incorporated elastic longitudinal coupling based on experimental data [28,43], the point stiffness values along the length presented in Fig 6A were 3–8 times greater than the stiffness per 10 μm section in Fig 6B. The OCC stiffness varied 1600 times along the cochlear length. While the BM stiffness varied most (a factor of 1700 between the basal and the apical ends), the TM axial and hair bundle’s linear stiffness varied much less (a factor of 110 and 47, respectively).

### The stiffness of the OCC imposed on the outer hair cell

**Fig 7:**
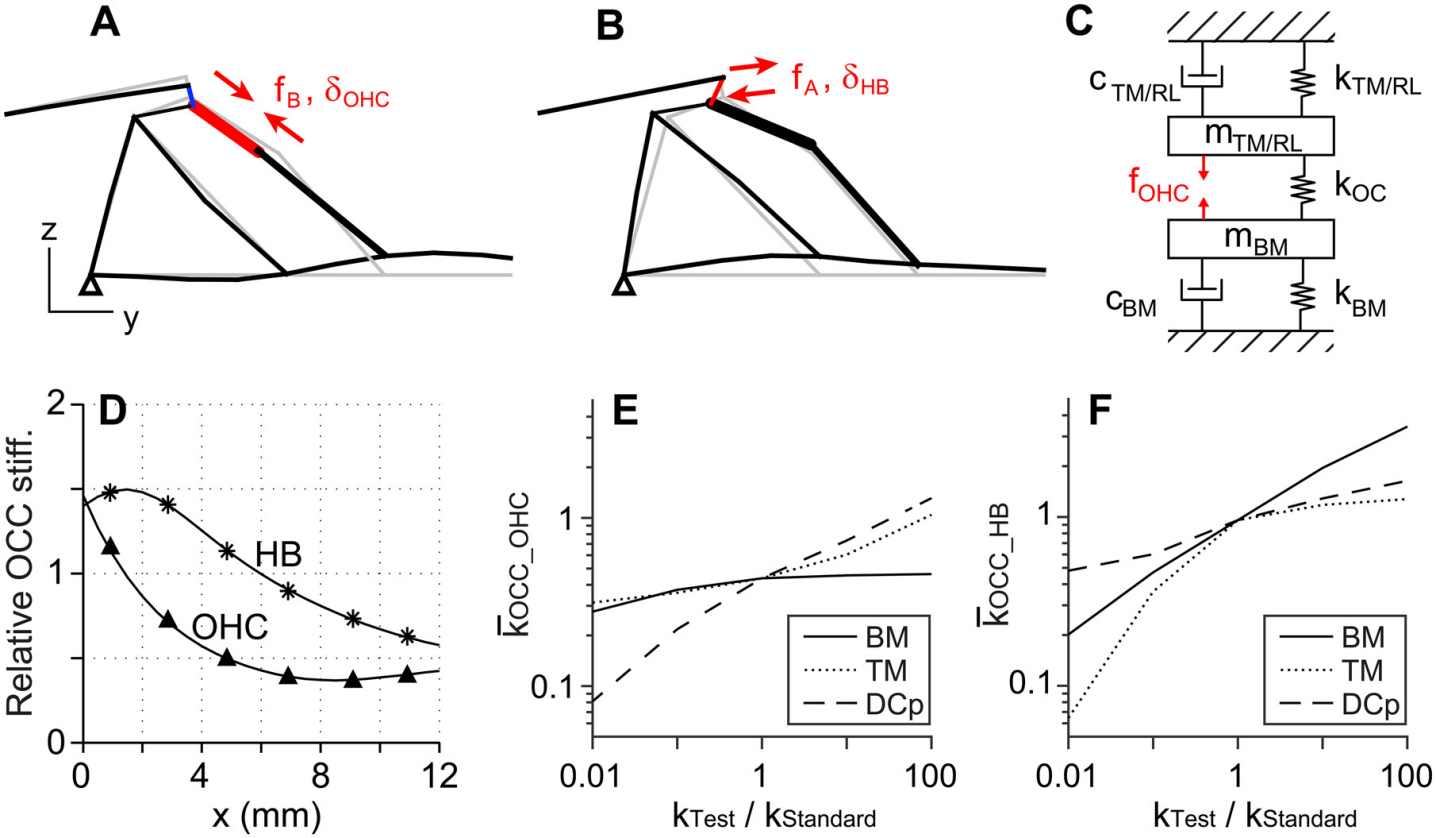
Elastic reactance experienced by the outer hair cell. **${\bar{k}}_{OCC\_OHC}$** and ${\bar{k}}_{OCC\_HB}$ were obtained by simulating the OCC deformation by reactive forces due to somatic motility and hair bundle motility. See text for the definition. (**A**) Deformation pattern of the OCC at *x* = 10 mm when a coupled axial force (*f*_*B*_) was applied to the outer hair cell. The corresponding displacement (*δ*_*OHC*_) represents the compliance of the OCC experienced by the outer hair cell. (**B**) Deformation pattern of the OCC at *x* = 10 mm when a coupled shear force (*f*_*A*_) was applied to the outer hair cell hair bundle. (**C**) In the conventional model of the OCC, the elastic reactance to the outer hair cell is determined by three components (*k*_*TM/RL*_, *k*_*OC*_, and *k*_*BM*_) that vary exponentially along the distance. (**D**) ${\bar{k}}_{OCC\_OHC}$ and ${\bar{k}}_{OCC\_HB}$ along the distance. (**E**) Change of ${\bar{k}}_{OCC\_OHC}$ due to the stiffness change of the BM, the TM and the Deiters cell. (**F**) Change of ${\bar{k}}_{OCC\_HB}$ due to the stiffness change of the BM, the TM and the Deiters cell. (E) and (F) results were obtained at *x* = 6 mm.

An important mechanical property related to the outer hair cell’s motility is the OCC stiffness experienced by the outer hair cells, or the elastic reactance imposed to the outer hair cells. To ascertain the elastic reactance acting on the two actuators of the outer hair cell (hair bundle and somatic motilities), coupled forces were applied at the top and the bottom nodes of the outer hair cell (*f*_*B*_, Fig 7A) or at the tip and the root of the hair bundle (*f*_*A*_, Fig 7B). When the resulting length change of the outer hair cell or the shear displacement of the hair bundle are denoted as *δ*_*OHC*_ or *δ*_*HB*_ respectively, the OCC elastic reactance normalized by the stiffness of each actuator (*k*_*OHC*_ or *k*_*HB*_) is

$${\bar{k}}_{OCC\_OHC}=k_{OCC\_OHC}/k_{OHC}$$ (10a)

$${\bar{k}}_{OCC\_HB}=k_{OCC\_OHB}/k_{HB}$$ (10b)

where,

$$k_{OCC\_OHC}=f_{B}/\delta_{OHC}-k_{OHC}$$ (11a)

$$k_{OCC\_HB}=f_{A}/\delta_{HB}-k_{HB}$$ (11b)

The elastic reactance to the outer hair cell, *K*_*OCC_OHC*_, changed by a factor of 13 throughout the cochlear length. When normalized with the stiffness of the outer hair cell at respective locations, the normalized elastic reactance to the outer hair cell,${\bar{k}}_{OCC\_OHC}$, ranged between 1.5 and 0.4 (Fig 7D). I.e., despite the 1600 times base-to-apex variation of OCC stiffness, the OCC stiffness felt by the outer hair cell remains comparable to the axial stiffness of the outer hair cell. Similarly, the OCC stiffness felt by the outer hair cell stereociliar bundle ranged between 1.5 and 0.6 times the hair bundle stiffness. This means that if an isolated outer hair cell can generate enough force to deform itself, the cell is also able to deflect the OCC.

This invariant elastic reactance to the outer hair cell is not the case with many existing studies using the lumped parameter model (Fig 7C). The *K*_*OCC_OHC*_ of this model is determined by the smaller stiffness between the upper structure representing the TM and the reticular lamina (*k*_*TM/RL*_) and the lower structure representing the BM (*k*_*BM*_) or

$$k_{OCC\_OHC,\;2DOF}=k_{TM/RL}k_{BM}/\left(k_{TM/RL}+k_{BM}\right).$$ (12)

In many cases, the ratio between the upper and the lower structure is constant, and the mechanical properties vary exponentially along the length as shown in Fig 6A. Therefore, in the two DOF models, K_OCC_OHC_ varies comparable to the BM stiffness.

The model framework like Fig 7C and Eq (12), that treats the upper structure (the TM or reticular lamina) and the lower structure (the BM) as elastic reactance in series to the outer hair cell actuator, agrees to the case of hair bundle force ($k_{OCC\_HB}$). When the shear force is applied through the hair bundle, the elastic reactance felt by the hair bundle actuator is well approximated by the lumped parameter model—i.e., ${\bar{k}}_{OCC\_HB}$ is determined by the more compliant component between the TM and the BM (Fig 7F). However, that is not the case with the outer hair cell somatic force. The somatic force deflects the BM in a complex pattern (Fig 7A) as was anticipated previously [44]. Unlike the elastic reactance to the hair bundle, the BM and the TM have very limited effect to *K*_*OCC_OHC*_ (Fig 7E). Instead, the Y-shaped structure formed by the outer hair cell, Deiters cell and its phalange affected *K*_*OCC_OHC*_ most.

### The effect of the Y-shaped structure in the organ of Corti

**Fig 8:**
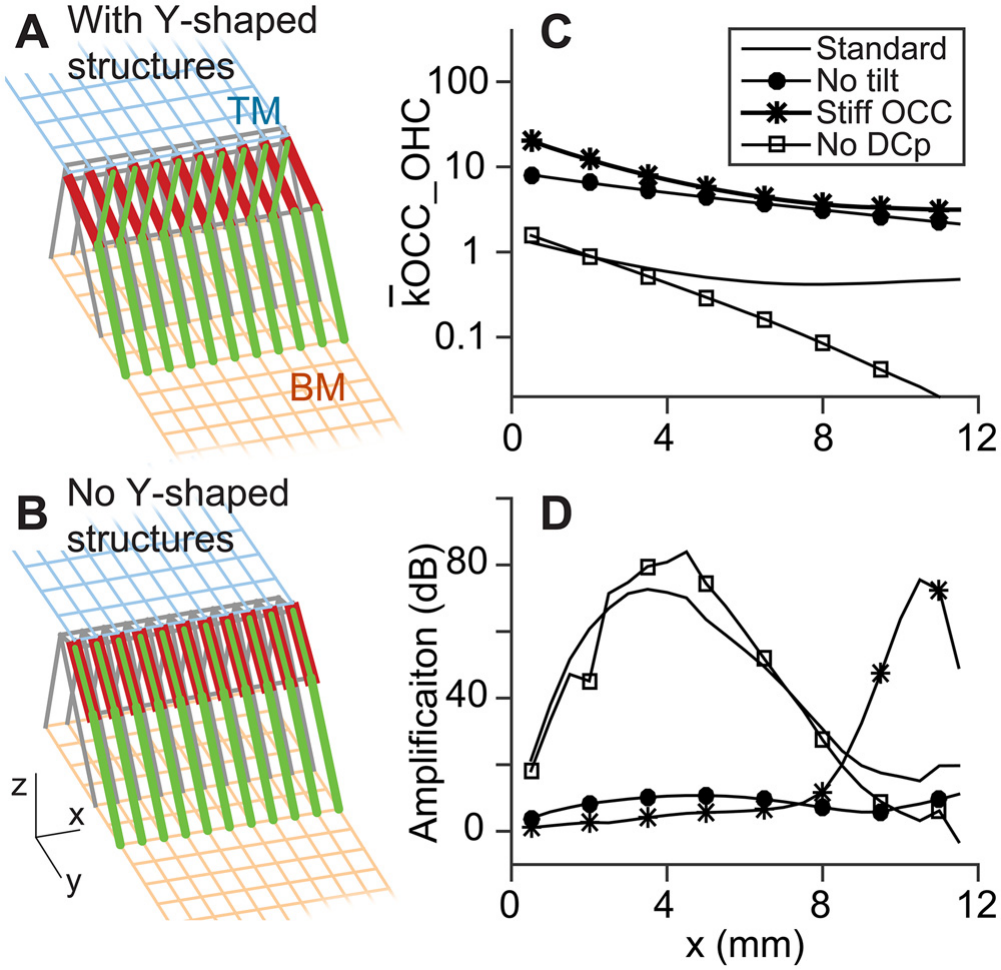
Effect of the Y-shaped structure formed by outer hair cell, Deiters cell and Deiters cell phalange. (**A**) The outer hair cells (red elements) tilt toward the basal direction while the Deiters cell phalanges (thin green elements) tilt in the opposite direction. Thus, they form repeating Y-shaped structures that behave like a truss structure. (**B**) To investigate the effect of the Y-shaped structure, either the tilt was set to zero (‘No tilt’ condition) or the Deiters cell phalanges were removed (‘No DCp’ condition). (**C**) The elastic reactance to the outer hair cell, ${\bar{k}}_{OCC\_OHC}$, increased by removing the tilt (‘No tilt’) or by increase the mechanical properties of the Deiters cell, its phalange and the reticular lamina (‘Stiff OCC’). ${\bar{k}}_{OCC\_OHC}$ decreased when there was no Deiters cell phalange. (**D**) The cochlear amplification was affected by the Y-shaped structures and the stiffness of the Deiters cell phalange.

The outer hair cells tilt toward the base and the Deiters cell phalange tilted toward the apex. Thus together with the Deiters cell body, they form repeating Y-shaped truss-like structures (Fig 8A). In such truss-like structures, mechanical loads transmit axially through each structural member. Thus the member’s axial stiffness is functionally more relevant than its bending stiffness unless the member buckles. With the standard parameters in Table 1, the axial stiffness of the outer hair cell was 47 and 19 mN/m at *x =* 2 and 10 mm, respectively. The axial stiffness of the Deiters cell phalange was greater than the outer hair cell by a factor of 2–5. When the stiffness of Deiters cell is much smaller or greater than the standard value, we could not find a working parameter set that ensures the amplification. Furthermore, the Y-shaped structure itself also affected the cochlear amplification.

To investigate how the Deiters cell morphology and stiffness affect cochlear mechanics, three cases were tested. First, ‘no-tilt’ case: The outer hair cell and the Deiters cell phalange were made upright in the x-z plane so that there was no Y-shaped structure (Fig 8B). The ${\bar{k}}_{OCC\_OHC}$ increased by 10 times (Fig 8C), and the amplification nearly disappeared. Second, ‘stiff OCC’ case: While the Y-shaped structures were in their original configuration, the Deiters cell, its phalange and the reticular lamina were stiffened until ${\bar{k}}_{OCC\_OHC}$ became similar to the first no-tilt case. The amplification disappeared between x = 0 and 8 mm, while for x > 9 mm, it was amplified much more than the standard case Third, ‘no DCp’ case: With all other conditions intact, the phalanges of the Deiters cells were removed. The amplification was comparable to the standard case between x = 0 and 8 mm. But, there was little amplification for x > 9 mm.

### Frequency-location relationship is strongly correlated with the passive mechanical properties

**Fig 9:**
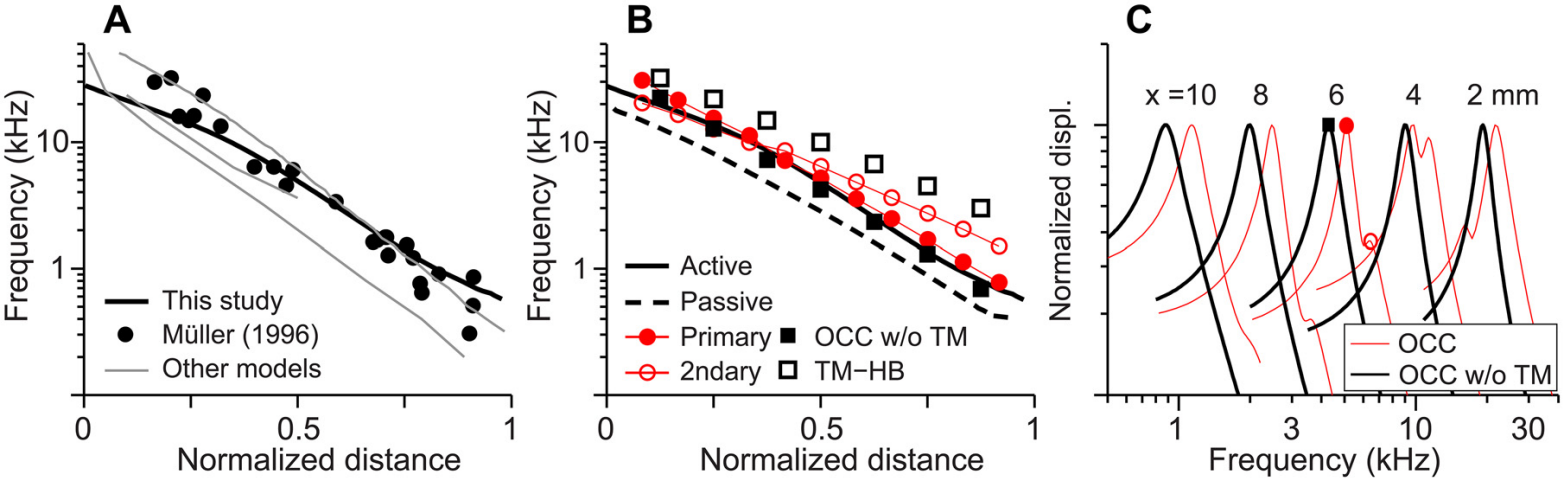
Frequency-location relations. (**A**) Frequency-location relationship of this study, a previous experiment [42] and other studies shown in Fig 6. (**B**) Comparison of whole cochlear model (active and passive cases) with the structural resonant frequencies of the OCC. (**C**) BM displacements to pure tone simulations of the OCC finite element model *without fluid-interaction*. The thick (black) curves are from the OCC without the TM and the thin (red) curves are from the enatic OCC model. The markers correspond to those in (B).

The simulated frequency-location relationship agreed with the experimentally measured relationship with some deviation at the basal end (Fig 9A, [42]). The active OCC’s best frequencies were higher than the passive values by 0.5–1 octave throughout the length of the cochlea (solid and broken curves, Fig 9B). In order to investigate how structural resonant frequencies compare to the hydro-dynamic cochlear frequencies, harmonic responses of the OCC finite element model without fluid-interaction or active outer hair cell feedback were analyzed. When pure tone stimulations were applied to the BM of the fluid-less OCC, there were two prominent peaks (thin curves, Fig 9C). However, when the TM was removed from the OCC, the secondary peak disappeared, which implies that the TM constituted a second resonator. The BM stiffness and the organ of Corti-BM complex mass makes the primary resonator and the mass of the TM and the stiffness of the TM-hair bundle complex results in the secondary resonator.

We investigated how the best frequencies of the two resonators compared to the best frequencies of the comprehensive OCC model incorporating the fluid-interaction and outer hair cell motility. The primary resonator’s best frequencies were obtained from harmonic analyses of the OCC finite element model after the removal of the TM. The secondary resonator’s best frequencies were analytically calculated from the mass of the TM and the sum of the TM axial stiffness and the hair bundle’s linear stiffness or *f*_2_ = (1/2π)((*k*_TM_+3*k*_HB_)/*m*_TM_)^0.5^. The structural best frequencies of the two resonators were shown together with the frequency-location curve of the whole model (including the fluid-interaction and outer hair cell motility) in Fig 9B. While both resonators’ best frequencies decreased toward the apex, the best frequency of the primary resonator was higher than that of the secondary resonator in the apex, but it was opposite in the apex. Overall the primary resonator’s best frequency was aligned well with the active comprehensive OCC’s best frequency. The reduction of the best frequency of the passive OCC is ascribed to the added fluid mass [45].

## Discussion

### Comparison with other studies

Our cochlear model reproduced characteristics that have been observed in cochlear micro-mechanical experiments. Using physiologically reasonable model parameters, the active OCC model achieved amplification and tuning levels (60 dB over the passive response and Q_10dB_ = 7.7 at *x* = 2 mm) that are comparable to experimentally observed values (e.g., [36,42]). The wavelength of the traveling waves ranged from 0.15 to 0.38 mm from the base to the apex when active and 0.8 to 1.6 mm when passive, which are comparable to experimental measurements of the gerbil cochlea [46]. Our model reproduced the characteristic phase difference between the OCC structures [47]. When active (equivalent to low sound-pressure-level stimulation), the vibrations of the top surface of the OCC led the bottom surface vibrations. This phase difference disappeared when passive (equivalent to high sound-pressure-level stimulation).

Our work confirmed some shared observations of previous models. Other theoretical models were summarized in Fig 6 and Table 2 for comparison. At least two mechanical DOF per OCC section were required to describe the differential motion of the top and bottom surfaces of the organ of Corti due to outer hair cell somatic motility. Despite some variations, all studies in Table 2 used equal or greater stiffness gradient than experimentally measured stiffness gradient (Fig 6A). Two previous cochlear models [14,21] incorporated both the hair bundle motility and somatic motility. The two studies showed that amplification characteristics were achieved primarily by the outer hair cell somatic motility, which agrees with this study.

Nam and Fettiplace [21] analyzed the phase difference between the OCC micro-structures, which is important to characterize the amplification by outer hair cell motility [23,48]. Their work had a reservation—it did not incorporate cochlear fluid dynamics. It was assumed in the study that intrinsic structural mechanics of the OCC plays an important role in cochlear mechanics regardless of fluid interaction. The proximity between the frequency-location curves of the comprehensive model and the structural model (OCC w/o TM in Fig 9) suggests that near highly tuned locations, the fluid acts as a pressure source rather than as an inertia. But, when passive (when poorly tuned), the fluid inertia contributed to the mechanics so much that the best frequency became lower than the structural resonant frequency by 0.5–1 octaves. This frequency shift was not observed without incorporating fluid dynamics. This study supports the primary conclusion of the previous work: the phase difference between the OCC structures is created by the outer hair cell somatic motility (Figs 4 and 5, [21]).

### Determinants of the steep frequency gradient in the cochlea

Despite the general agreement that the frequency-place relationship of the cochlea is determined mechanically, there is less agreement on which mechanical attributes determine the frequency gradient. For example, Naidu and Mountain [24] measured 5.6 dB of stiffness change per octave frequency change to conclude that the stiffness gradient of the OCC is not sufficient to explain the frequency gradient, while Emadi and his colleagues [4] obtained 8.3 dB/octave to conclude the opposite (these gradient values were obtained after accounting for the longitudinal space constant, S1 Supporting Theory). We used the steeper stiffness gradient of the two experiments to obtain our results (Fig 6A). With the smaller stiffness gradient, we could not achieve the physiological frequency range for the modeled species. All the models in our Table 1 (though not an exhaustive list of theoretical models) used equal or greater stiffness gradient than the experiments. While most studies used a base-to-apex mass ratio (m_base_/m_apex_) between 0.1 and 0.3 based on the cross-sectional area of the OCC, one study used about 0.01 [15] and another study used greater than one [49]. Among theoretical studies, despite some variation, the gradient of the structural resonance frequency corresponds well with the slope of the physiological frequency.

A recent study [50] reproduced the characteristic cochlear traveling waves without mechanical resonance (i.e., no structural mass was incorporated). The model achieved the frequency gradient through the gradients of the fluid inertia and the OCC stiffness. The fluid inertia, proportional to the square of the wavelength of the traveling wave, does not have a steep gradient. The frequency gradient of their gerbil cochlea model was achieved by the stiffness gradient (the base-to-apex ratio of 60,000). Perhaps, the gist of the work was to propose an alternative physical principle of the cochlear traveling wave generation, not to address the origin of the frequency gradient. The author demonstrated that the interference between different propagation modes along the top and bottom surfaces of the OCC can create propagating waves similar to cochlear traveling waves, which contrasts to the popular theory of the critical layer absorption [45]. Note that the theory of critical layer absorption requires mechanical resonators with a graded resonant frequency to arrest the traveling waves.

Our model obtained the frequency gradient through three mechanical components: structural mass and stiffness, and fluid inertia. Two structural resonant frequencies were prominent—one originating from the BM-organ of Corti complex and the other from the TM and the hair bundles of the outer hair cells. When active, the fluid inertia became smaller than the structural mass and the best frequency was well-approximated by the resonance frequency of the BM-organ of Corti complex. When passive, the fluid inertia shifted the frequency by 0.5 to 1.0 octaves lower. Like most other existing theoretical models, the BM stiffness gradient was the pivotal component that determines the frequency gradient of our model. It is worthwhile noting that, with our continuum mechanical model, unlike lumped parametric models, the *effective* mass of the OCC structure depends on the vibration mode. Out-of-phase vibrations between the active OCC structures were observed in contrast to in-phase vibrations of the passive OCC (Figs 4 and 5). In other words, depending on the vibration modes, our continuum mechanical model redistributes the structural mass between the two resonators.

### Location-dependence of OCC mechanical responses

Our model predicted two qualitative differences between the basal and the apical OCC mechanical responses. First, the top surface of the OCC (represented by the TM) vibrated with greater displacement amplitude than the bottom surface (represented by the BM) in the basal locations, but opposite was true in the apical locations (Fig 3B and 3C). The outer hair cell motility increased the difference further. Similar responses were observed experimentally. According to Chen et al.’s measurements [47], the reticular lamina-BM displacement ratio at the basal turn was 1.8 and 2.6 at high and low sound pressure levels, respectively. These values are comparable to our simulated TM-BM displacement ratio of 1.3 (passive) and 2.0 (active) at *x* = 2 mm (Fig 4). Second, the TM vibrated with a higher mode in the base while the BM vibrated with a higher mode in the apex (Fig 5). Different modes of TM vibration were observed experimentally [51,52], and their consequences to inner hair cell’s mechano-transduction were speculated on [37]. At the basal locations, a similar BM vibration pattern as ours was observed experimentally [53] and analyzed with a finite element model [54]. But, location-dependent TM or BM vibration modes have not been observed in experiments.

Different mechanical contributions of the BM and other OCC structures that depend on longitudinal location are responsible for the location-dependence of OCC mechanical responses. The BM dominates the stiffness gradient of the OCC. In the present study, the BM stiffness was determined by the thickness of the collagen fiber layer in the pectinate zone, and the width of the BM, which vary by factors of 0.22 and 1.75, respectively, between *x* = 2 mm and 10 mm. The Young’s modulus of the BM was kept constant throughout the cochlear length (1 GPa, based on the value of collagen fibers). Unlike the BM whose flexural rigidity represents its mechanical stiffness, the stiffness of other OCC structures was better represented by their axial rigidity because of their truss-like arrangement. The stiffness due to flexural rigidity is proportional to *b* ^*4*^ */L* ^3^, where *b* and *L* are the thickness and length of the structure, respectively, while the stiffness due to axial rigidity is proportional to *b* ^*2*^ */L*. Furthermore, the thicknesses of the OCC structures such as the pillar cells, Deiters cells and outer hair cells hardly change along the cochlear length, and there is no evidence that their elastic moduli change along the cochlear length either. As a result, in contrast to the BM stiffness, the stiffness of truss-like OCC structures change very modestly along the length of the cochlea. The TM and the hair bundle might be the only other structures whose flexural rigidity may contribute to the OCC mechanics (as was considered in [55]). Within our investigated range of TM Young’s modulus (between 1 kPa and 10 MPa, radial direction), the TM’s flexural rigidity had a much less effect on OCC mechanics than its axial rigidity. With the standard material properties, the TM’s axial stiffness is more than a hundred times greater than its cantilever stiffness (Fig 6B). To conclude, because of the steep stiffness gradient of the BM, the BM stiffness dominates the OCC stiffness in the basal end, but the other OCC structures contribute to overall OCC stiffness more than the BM in the apex (Fig 6B). This difference primarily explains the location-dependence of the OCC mechanical responses that were observed in this study.

Because of the similarity between neural and mechanical tuning curves [56], the BM displacement has been assumed to faithfully represent the neural responses. Often the amplification of BM displacements and the cochlear amplification are used interchangeably. But, to be accurate, the purpose of the cochlear amplifier is to amplify the inner hair cells’ neural responses rather than the BM displacements. Although our model did not incorporate inner hair cells explicitly, the outer hair cell’s hair bundle displacement or the TM displacement must better predict the inner hair cell responses than the BM displacement. Our results suggest that 1) the OCC mechanical gain (defined as the hair bundle displacement per BM displacement) will be location-dependent (Fig 4C) and 2) the BM displacement and the neural tuning curves can be different at low frequency locations (Fig 4A).

### Location-dependent contributions of the Deiters cell to cochlear amplification

We found that the Y-shaped structure formed by the outer hair cell and the Deiters cell plays a crucial role in the OCC mechanics (Fig 7) and the cochlear amplification (Fig 8). In the apex, the Y-shaped structure provides a proper structural rigidity for the outer hair cells to deliver the reactive force to the TM and the BM. Without the Deiters cell phalange, the OCC was too compliant (${\bar{k}}_{OCC\_OHC}$ was too small) to deliver the somatic force to the TM or the BM. In the base, the Y-shaped structure couples the top (the TM) and the bottom (the BM) oscillators so that the two surfaces carry cooperatively interacting traveling waves. Without the Deiters cell phalange, we observed two sharp peaks in high frequency (> 10 kHz) tuning curves—one by the BM and the other by the TM.

### Stiffness of the OCC felt by the outer hair cells is equalized throughout the cochlea

A concern regarding the outer hair cell’s contribution to cochlear amplification is that, because the OCC becomes stiffer toward the basal end, the outer hair cells may need greater force to deform the OCC [27]. Indeed, previous cochlear models needed a greater active force of the outer hair cell toward the base [49,57–59]. It is rather exceptional to use a constant outer hair cell force (e.g., the models of Grosh and his colleagues [14,55]). Our model used physiologically grounded outer hair cell force that is nearly constant or even decrease toward the base. Despite the constant electro-mechanical gain of the outer hair cell (0.1 nN/mV, [26]) and the limiting RC time constant of the outer hair cell’s membrane (filtering frequency of 0.4 kHz and 7.3 kHz at *x* = 2 mm and 10 mm, respectively, [19,21]), our model could amplify at the stiff basal location more effectively than at the compliant apex. How does the cochlea resolve the mismatch between the steep OCC stiffness gradient and the flat outer hair cell active force?

What matters for the outer hair cell actuator is not the OCC stiffness opposing the fluid pressure, but the OCC stiffness imposed on the outer hair cells. According to our continuum mechanics-based OCC model, the stiffness felt by the outer hair cell does not vary much along the cochlear length. While the OCC stiffness changed by a factor of 1600, the elastic reactance of the OCC imposed on the outer hair cell varied only by a factor of 7. The OCC elastic reactance ranges between 1.5 and 0.4 times the outer hair cell stiffness throughout the cochlear length. Our work reveals an operating condition for the outer hair cells to be an efficient cochlear amplifier—equalized elastic reactance to the outer hair cells throughout the cochlea.

Our results indicate that the hair bundle force has limited influence for the amplification of OCC vibrations. But, in a previous study [28], it was shown that the hair bundle force, albeit small (at most 10–100 pN per hair cell), can deform the OCC effectively. The elastic reactance of the OCC imposed on the hair bundle was also equalized throughout the cochlear length (Fig 7D), which suggests that the hair bundle force also has the capability to modulate the OCC mechanics. Although we did not observe a clear effect of the hair bundle motility, there may be conditions under which the hair bundle motility can affect the OCC mechanics. For example, a recent work [60] showed that the viscous damping in the sub-TM space is frequency-dependent and the hair bundle motility can modify the viscous damping versus frequency relationship. Although such an adaptive damping mechanism was not incorporated in this work, we cannot exclude the interesting possibility that the hair bundles of outer hair cell and the inner hair cell may interplay to contribute the cochlear mechanics by modulating the energy dissipation in the sub-TM space.

### Two fluid-interacting surfaces

Considering the BM as a single fluid interacting surface has been a standard approach. As a result, the fluid pressures at the top and the bottom fluid domains are considered anti-symmetrical (equal and opposite). But, two lines of experimental evidence are not consistent with the standard approach—single fluid interacting surface. First, the top and the bottom surfaces of the OCC do not vibrate in phase when the sound pressure level is low (< 80 dB) [47,61]. This implies that, in theory, two elastic media (the TM and the BM) can carry different traveling waves [62,63]. The second evidence is more direct—the pressures measured in the scala vestibuli and the scalar tympani are not anit-symmetric [64].

Recent studies [41,50,65] investigated the dual traveling waves along the top and the bottom surfaces of the OCC to show that a finite elastic coupling between the two surfaces is required to form dual traveling waves. Our model is in line with these studies in that the model has two fluid-interacting surfaces and the two surfaces are coupled with elastic structure(s).

**Fig 10:**
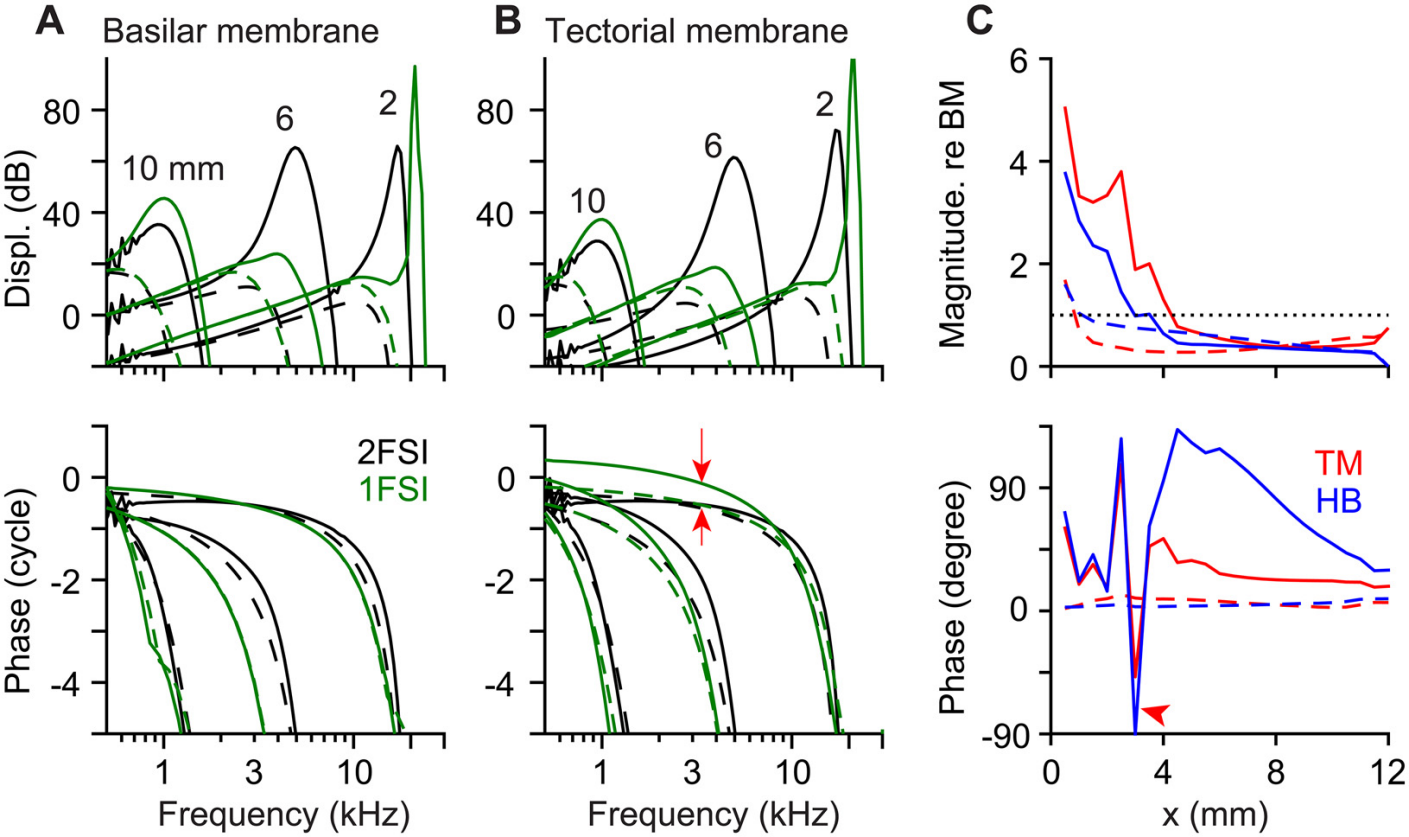
Two versus one-fluid interacting surface. (**A**) BM displacement amplitude and phase along the stimulating frequency. Solid/broken lines indicate active/passive responses. Black lines correspond to two fluid-interacting surface case, while green lines to single fluid-interacting surface case. (**B**) TM displacement and phase. (**C**) Relative motion of the TM (red) and the hair bundle (blue) with respect to the BM motion.

The responses when there was single fluid-interacting surface (the BM) were compared with the standard case in Fig 10. Although the overall trends of the two cases were similar, there were several notable differences. With the single interacting surface: The amplification and tuning in the middle turn were reduced. The 180 degree phase reversal between x = 3 and 4 mm (arrow head in Fig 10C) seems to be responsible for the minimal amplification. There was a half cycle phase difference between the active and the passive TM responses (arrow in Fig 10B). Unlike the standard case, the fluid pressures in the upper and the lower fluid space were anti-symmetric (Fig D in S1 Supporting Theory).

Even though we present Fig 10, we are hesitant to make conclusions regarding two fluid-interacting surfaces because we haven’t explored the effect thoroughly. For example, when reducing the 3-D fluid space into a 2-D model, the midpoint of the BM is reasonable to represent the bottom interacting surface. Although we chose a point along the TM above the outer hair cell’s hair bundle, the top interacting surface is ambiguous. A full 3-D simulation of the fluid domains will provide clearer insights. Also more exhaustive parametrical study is necessary to test if the observed characteristics of the two fluid-interacting model are physiologically meaningful or not.

## Supporting Information

S1 Movie Clip An animation of vibrating organ of Corti. (WMV)

S1 Supporting Theory Detailed derivation procedures with a table and four figures. (DOCX)
