# Supplementary material for: Consequences of Location-Dependent Organ of Corti Micro-Mechanics
Source: PLoS One. 2015 Aug 28;10(8):e0133284. doi: 10.1371/journal.pone.0133284 (PMC4552730; doi:10.1371/journal.pone.0133284)
Supplement: S1 Supporting Theory — (DOCX) [file pone.0133284.s002.docx]

# Supporting Information

**Title: Consequences of location-dependent organ of Corti micro-mechanics**

**Authors:** Yanju Liu^1^, Sheryl M. Gracewski^1, 2^, and Jong-Hoon Nam^1, 2^

**Affiliation:**

^1^Department of Mechanical Engineering, ^2^Department of Biomedical Engineering

University of Rochester, Rochester, NY 14627, USA

## Governing equation and boundary conditions of the fluid domain

The 2D Navier-Stokes equations were reduced to Laplace equation, such that

$\frac{\partial^{2}p}{\partial x^{2}}+\frac{\partial^{2}p}{\partial z^{2}}=0,$ for $0<x<L, -H<z<H$ (A)

where, $p$ is the pressure, $L$ is the length of cochlea and $H$ is the height of the fluid space above and below the OCC. For the modeled gerbil cochlea, *L* = 12.1 mm and *H* = 0.3 mm. The boundary conditions for the fluid domain are

$\left\{ \begin{matrix} \begin{matrix} \partial p/\partial x= -2\rho a_{S},\mathrm{at} x=0, 0<z<H \\ p=0,\mathrm{at} x=0,-H<z<0 \\ \partial p/\partial x=0, \mathrm{at} x\geq L-c \end{matrix} \\ \begin{matrix} \partial p/\partial z=0, \mathrm{at} z=\pm H \\ \partial p/\partial z=-\rho a_{CP}^{+}, \mathrm{at} z=+0 \\ \partial p/\partial z=-\rho a_{CP}^{-}, \mathrm{at} z=-0 , \end{matrix} \end{matrix} \right.$ (B)

where $\rho$ is the density of the cochlear fluid, $a_{S}$ is the acceleration of the stapes, $a_{CP}^{+}$ and $a_{CP}^{-}$ are the accelerations of the top and bottom surfaces of the OCC, respectively, and *c* is the span of the helicotrema (*c* = 0.1 mm).

The governing equation, Eq. (S1), is discretized according to the finite difference scheme. The discretized governing equation including the boundary conditions are

$$\left\{ \begin{matrix} p_{1,n-1}-4p_{1,n}+p_{1,n+1}+2p_{2,n}=-2\rho dxa_{S}, m=1, n=nCP+1,\cdots N \\ 2p_{1,N}-4p_{1,N+1}+{2p}_{2,N+1}=0, m=1,n=N+1 \\ p_{1,n}=0, m=1, n=2,3,\cdots,nCP-1 \\ {2p}_{1,2}-4p_{1,1}+{2p}_{2,1}=0, m=1,n=1 \\ p_{m-1,N+1}+2p_{m,N}-4p_{m,N+1}+p_{m+1,N+1}=0, m=2,3,\cdots,M,n=N+1 \\ {{2p}_{M,N+1}+2p}_{M+1,N}-4p_{M+1,N+1}=0, m=M+1,n=N+1 \\ 2p_{M,n}+p_{M+1,n-1}-4p_{M+1,n}+p_{M+1,n+1}=0, m=M+1,n=2,3,\cdots,N \\ 2p_{M,1}+2p_{M+1,2}-4p_{M+1,1}=0, m=M+1,n=1 \\ p_{m-1,1}-4p_{m,1}+2p_{m,2}+p_{m+1,1}=0, m=2,3,\cdots,M,n=1 \\ p_{m-1,nCP-1}+2p_{m,nCP-2}-4p_{m,nCP-1}+p_{m+1,nCP-1}-2\rho\Delta zi\omega V_{m,nCP-1}=0, m=2, 3,\cdots,M, n=nCP-1 \\ p_{m-1,nCP+1}-4p_{m,nCP+1}+2p_{m,nCP+2}+p_{m+1,nCP+1}-2\rho\Delta zi\omega V_{m,nCP+1}=0, m=2, 3,\cdots,M, n=nCP+1 \end{matrix} \right.$$

(C)

where $m=1, m=M+1, n=1, \mathrm{and} n=N+1$ correspond to the left, right, bottom and top boundaries, respectively, and $n=nCP\pm1$ represents the top and the bottom surfaces of the OCC.

## Linearization of outer hair cell transmembrane potential

The outer hair cell somatic motility and its hair bundle’s mechano-transduction were represented with nonlinearity incorporated. In the current study, we assume that with small harmonic perturbations around a resting point of the system, outer hair cell lateral membrane conductance and capacitance are linearly dependent on its transmembrane potential. The outer hair cell receptor potential can be solved by the following equation along time

$\left(C_{m}+C_{s}+\alpha\right)dV_{m}/dt=G_{s}\left(E_{e}-V_{m}\right)-G_{m}\left(V_{m}+E_{K}\right)$ (D)

where $C_{m}$and $G_{m}$are the outer hair cell’s membrane capacitance and conductance, $C_{s}$and $G_{s}$the outer hair cell’s stereocilium capacitance and conductance, $E_{e}$the endocochlear potential, $E_{K}$ the outer hair cell equilibrium potential dominated by the potassium equilibrium potential, and $\alpha$ is defined as$\alpha=V_{m}(dC_{m}/dV_{m})$. This equation describes how outer hair cell’s transmembrane potential $V_{m}$changes in response to the change in mechano-transduction current or, equivalently$p_{o}$, the open probability. To linearize, a small harmonic perturbation in the open probability of the magnitude $\delta p_{o}$about the resting open probability$p_{o}^{0}$ is considered. This small perturbation causes a change in transmembrane potential and stereocilia conductance.

$\left\{\begin{matrix} G_{s}=G_{s}^{\max}(p_{o}^{0}+\delta p_{o}e^{i\omega t}) \\ V_{m}=V_{m}^{0}+\delta V_{m}e^{i\omega t} \end{matrix}\right.$ (E)

where$G_{s}^{\max}$ is the maximum conductance of a hair bundle. The membrane conductance and capacitance are dependent on the membrane potential.

$\left\{\begin{matrix} C_{m}=C_{m}^{0}+s_{C}^{0}\delta V_{m}e^{i\omega t} \\ G_{m}=G_{m}^{0}+s_{G}^{0}\delta V_{m}e^{i\omega t} \\ \alpha=\alpha_{0}+s_{\alpha}^{0}\delta V_{m}e^{i\omega t} \end{matrix}\right.$ (F)

where $\left.s_{C}^{0}=(dC_{m}/dV_{m})\right|_{V_{m}^{0}}$, $s_{G}^{0}=\left.(dG_{m}/dV_{m})\right|_{V_{m}^{0}}$, and $s_{\alpha}^{0}=\left.(d\alpha/dV_{m})\right|_{V_{m}^{0}}$ are the slope of change of $C_{m}$, $G_{m}$, and α with $V_{m}$, respectively. The resting open probability, transmembrane potential is also at rest is

$G_{s}^{0}\left(E_{e}-V_{m}^{0}\right)-G_{m}\left(V_{m}^{0}+E_{K}\right)=0$. (G)

From Eq. (S4-7), after neglecting terms contains higher order terms, we obtain

$\delta V_{m}=\delta p_{o}G_{s}^{\max}(E_{e}-V_{m}^{0})/\left(G_{s}^{\max}p_{o}^{0}+G_{m}+s_{G}^{0}E_{K}+i\omega(C_{m}+C_{s}+\alpha_{0})\right)$. (H)

The voltage-dependence terms $s_{G}^{0}$ and $\alpha_{0}$ are (3.6, 0.3) nS/mV and (-2.1, 1.1) pF for x = (2, 10) mm, respectively. The other parameter values are presented in Table 1. The values at other locations were interpolated/extrapolated from these two sets of parameter values such as,

$C_{s}\left(x\right)=C_{s}\left(x_{0}\right)e^{a_{1}\left(x-x_{0}\right)}$, $a_{1}=\log\left(C_{s}\left(x_{1}\right)-C_{s}\left(x_{0}\right)\right)/\left(x_{1}-x_{0}\right)$. (I)

## Linearization of mechano-transduction channel kinetics and hair bundle mechanics

Our model assumes a mechano-transduction channel to have four Ca^2+^-binding sites and two configurations (open or closed, Fig S1). Using the constraint$\sum_{i=0}^{9} p_{i}=1$ to express one state probability as a function of others as$p_{1}=1-\sum_{i=2}^{9} p_{i}$, the equations of the kinetic scheme of the 10-state channel can be reduced to the nonsingular form

, (J)

where $\hat{\mathbf{p}}$ is the vector column of state probabilities (the hat symbol is to distinguish from the pressure symbol), **A** is 9 × 9 coefficient matrix that is a function of stereocilia bundle displacement *ξ_HB_,* and **B** is a column vector. Matrices **A** and **B** are

$$\mathbf{A}=- \left[\begin{matrix} k_{21}+k_{23}+k_{29}-k_{12} & k_{12}-k_{32} & k_{12} & k_{12} & k_{12} & k_{12} & k_{12} & k_{12}-k_{92} & k_{12} \\ -k_{23} & k_{32}+k_{34}+k_{38} & -k_{34} & 0 & 0 & 0 & -k_{83} & 0 & 0 \\ 0 & -k_{34} & k_{43}+k_{45}+k_{47} & -k_{54} & 0 & -k_{74} & 0 & 0 & 0 \\ 0 & 0 & -k_{45} & k_{54}+k_{56} & -k_{65} & 0 & 0 & 0 & 0 \\ 0 & 0 & 0 & -k_{56} & k_{56}+k_{67} & -k_{76} & 0 & 0 & 0 \\ 0 & 0 & -k_{47} & 0 & -k_{67} & k_{74}+k_{76}+k_{78} & -k_{87} & 0 & 0 \\ 0 & -k_{38} & 0 & 0 & 0 & -k_{78} & k_{83}+k_{87}+k_{89} & -k_{98} & 0 \\ -k_{29} & 0 & 0 & 0 & 0 & 0 & -k_{89} & k_{92}+k_{98}+k_{910} & -k_{09} \\ k_{10} & k_{10} & k_{10} & k_{10} & k_{10} & k_{10} & k_{10} & k_{10}-k_{90} & k_{01}+k_{09}+k_{10} \end{matrix}\right]$$

(K)

$\mathbf{B}=\left[\begin{matrix} -k_{12} & 0 & 0 & 0 & 0 & 0 & 0 & 0 & -k_{10} \end{matrix}\right]^{T}$ (L)

|  | **Figure A**. **Mechano-transduction channel kinetics**. (**A**) The channel has closed (C) or open (O) configuration, and four calcium binding sites. The four binding sites have the same affinity. (**B**) State diagram with ten state variables from p_1_, p_2_, ···, p_0_, which correspond to the probability of staying at the state of C, CCa, ···O. In this illustration, the areas of circles are proportional to the probability of individual states. This figure is from Prodanovic et al. [[1](#_ENREF_1)] with the permission from the authors. |
| --- | --- |

Where, the $\hat{\mathbf{p}}=\left[\begin{matrix} p_{2} & p_{3} & p_{4} & p_{5} & p_{6} & p_{7} & p_{8} & p_{9} & p_{0} \end{matrix}\right]^{T}$. The superscript T is the transpose operator. Opening and closing rates, *k_OC_* and *k_CO_* in Fig. S1 are given by

$\left\{\begin{matrix} k_{CO}=k_{F}\exp(\Delta E/2k_{B}T) \\ k_{OC}=k_{F}\exp(-\Delta E/2k_{B}T) \end{matrix}\right.$ (M)

and Ca-binding and unbinding rates, $k_{Ca}^{+}$ and $k_{Ca}^{-}$ by

$\left\{\begin{matrix} k_{Ca}^{+}=k_{B}C_{FA} \\ k_{Ca}^{-}=k_{B}K_{D} \end{matrix}\right.$, (N)

where *k_b_* is calcium binding coefficient, *C_FA_* is calcium concentration at binding site and *K_D_* is calcium dissociation constant *k_B_* is Boltzmann constant, *T* is the absolute temperature and *k_F_* is the opening/closing rate constant. In the model, $k_{B}T=4\times{10}^{-21}J$. The term *ΔE* represents the difference in the channel intrinsic energy between open and closed states that depends on the hair bundle displacement, gating swing, and the number of bound calcium ions as

$\Delta E=\gamma b k_{G}(\xi_{HB}-\xi_{S}-n_{Ca}b_{FA})$, (O)

where *γ* is the geometric gain, *b* is the gating swing, *k_G_* is the gating spring stiffness, *n_Ca_* is number of bound calcium ions, *b_FA_* is the coefficient to incorporate the effect of calcium-dependent adaptation and $\xi_{S}$ is a constant used to set the resting open probability. In this study, the resting open probability is 0.4.

At equilibrium,

$\mathbf{A}_{0}\hat{\mathbf{p}}_{0}+\mathbf{B}_{0}=0$, (P)

where the subscript 0 denotes the parameter is evaluated at equilibrium. Now, the Eq. (S11) can be linearized about the equilibrium position by taking Taylor expansion of matrix **A** and vector **B.**

$\mathbf{A}=\mathbf{A}_{0}+\mathbf{A}'_{0}\left(\xi_{HB}-\xi_{HB}^{0}\right)$ (Q)

$\mathbf{B}=\mathbf{B}_{0}+\mathbf{B}'_{0}\left(\xi_{HB}-\xi_{HB}^{0}\right)$ (R)

Substituting the above two equations into the Eq. (S10) yields linearized equation of the channel kinetics

(S)

By assuming small perturbations around the equilibrium point in the form of time harmonic variations

$\xi_{HB}=\xi_{HB}^{0}+\delta\xi_{HB}e^{i\omega t}$ (T)

(U)

From Eq. (S19-21),

(V)

After neglecting higher order terms,

. (W)

The hair bundle deflection, $\delta\xi_{HB}$, is give from the finite element model. By defining $\xi_{HB}^{0}=0,$ $\xi_{HB}=\delta\xi_{HB}$. The model parameters related to the hair bundle’s mechano-transduction are in Table S1.

| **Table A**. **OHC Hair bundle mechano-transduction parameters** | | | | | |
| --- | --- | --- | --- | --- | --- |
| Symbol | | Apex | Base | Description | Reference |
| *γ* | - | 0.1 | 0.25 | Elongation of the gating spring per unit displacement of hair bundle tip | [[2](#_ENREF_2),[3](#_ENREF_3)] |
| *k_F_* | (ms^-1^) | 100 | 100 | Channel activation rate constant | [[2](#_ENREF_2),[3](#_ENREF_3)] |
| $k_{GS}$ | (mN/m) | 6 | 6 | Gating spring stiffness | [[2](#_ENREF_2),[3](#_ENREF_3)] |
| *b* | (nm) | 0.5 | 0.5 | Gating swing | [[2](#_ENREF_2),[3](#_ENREF_3)] |
| *N* |  | 60 | 75 | Number of transduction channels | [[2](#_ENREF_2),[3](#_ENREF_3)] |
| $\xi_{S}$ | (nm) | 1.5 | 0.6 | Setting point of mechano-transduction |  |
| *b_FA_* | (nm) | 0.7 | 0.7 | Ca binding modification | [[2](#_ENREF_2),[3](#_ENREF_3)] |
| *k_B_* | (ms^-1^µM^-1^) | 0.4 | 0.4 | Ca binding coefficient | [[2](#_ENREF_2),[3](#_ENREF_3)] |
| *K_D_* | (µM) | 40 | 100 | Ca dissoc. const. when a channel is closed | [[2](#_ENREF_2),[3](#_ENREF_3)] |
|  |  | 1 | 1 | Ca dissoc. const. when a channel is open | [[2](#_ENREF_2),[3](#_ENREF_3)] |
| *C*_FA_ | (µM) | 1 | 1 | [Ca^2+^] near the channel when a channel remains closed | [[4](#_ENREF_4),[5](#_ENREF_5)] |
|  |  | 40 | 100 | [Ca^2+^] near the channel when a channel remains open | [[4](#_ENREF_4),[5](#_ENREF_5)] |

## Parametric study of the TM Young’s modulus

| 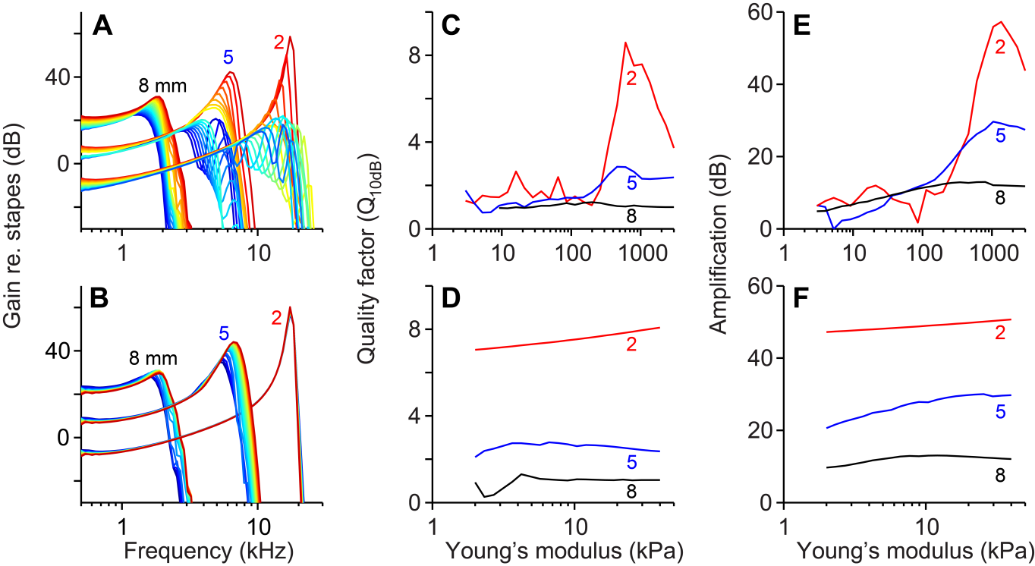 |
| --- |
| **Figure B. Parametric study of TM body Young’s modulus. (A)**  Frequency responses of BM displacement at the base, middle and apex relative to stapes when varying TM body Young’s modulus in the base (hotter color corresponds to greater TM modulus). **(B)**  Frequency responses of BM displacement when varying TM body Young’s modulus in the the apex. **(C)** The quality factor, Q_10dB_ at best frequency at base (red), middle (blue) and apex (black) predicted by model as a function of TM body Young’s modulus in the base varies between 3 kPa and 3 MPa **(D)** The quality factor as the apical TM modulus varies between 2 to 40 kPa. (**E**) Amplification in dB at best frequency when varying TM body Young’s modulus in the base between 3 kPa to 3 MPa and **(F)** Amplification in dB at best frequency when varying TM body Young’s modulus in the apex between 2 to 40 kPa. |

TM mechanical properties have been shown to affect cochlear frequency tuning and amplification [[6](#_ENREF_6)]. The measured Young’s modulus of the TM in the literature varies by more than two orders of magnitude depending on preparations and probing methods. Furthermore, whether there is a significant base-to-apex longitudinal gradient for TM Young’s modulus is unclear (see [[7](#_ENREF_7)] for a summary). In the TM, collagen fibers aligned along the radial direction, which are interconnected by different fibers and the bottom surface of the TM is covered with a net-like structure. In this work, the anisotropy of the TM was represented by a meshwork of beam elements running in the radial and longitudinal directions, with distinct elastic moduli. To determine an optimal set of values of for the TM Young’s modulus in the radial direction, we have varied the TM Young’s modulus from 3 kPa to 3 MPa for the base, and 2 kPa to 40 kPa for the apex (Fig. S2). When varying the basal values while holding apical value constant (Fig. S2, top panels), the quality factor of the BM’s mechanical response and active gain both reached a maximum around 800 kPa. Depending on the TM elastic properties, the best frequency at *x* = 2 mm varies between 10 to 30 kHz. At the basal location, when the TM is compliant (cool colored curves, Fig. S2 top panels), the BM motion has 2 peaks and was affected by the second mode of the TM bending deformation. As TM became stiffer, two response peaks merge into one. When the apical values were varied while the basal value kept constant, the best frequency, the mechanical quality factor and the active were minimally affected by the 10-fold of change of TM Young’s modulus (Fig. S2, bottom panels).

To summarize, there existed an optimal TM Young’s modulus that resulted in the tuning quality and amplification that are comparable to experimentally measured values. The highest quality factor and the greatest amplification was achieved near basal TM Young’s modulus of (800, 40) kPa at x = (2, 10) mm.

## Conversion of stiffness in previous modeling studies to stiffness per section

In the literature of cochlear models, stiffness (and mass) parameter is usually expressed in terms of stiffness per unit CP area (also called volumetric stiffness) in order to convert from mechanical impedance to acoustical impedance. However, it can also be in stiffness per unit length in other models, for example, finite element models [[8](#_ENREF_8)], or other lumped-parameter models [[9](#_ENREF_9)]. To compare stiffness among literature, we will need to convert them into the same unit system. In Table S2 we showed what was presented in the original studies and the values after conversion into stiffness per 10 μm CP section. Here, we show how conversion were done generally and will also discuss entries in Table S2 that need special attention.

Generally, if stiffness values are presented in per unit CP area, k_A_, we multiply k_A_ by the area of a 10 μm section (10 times width of BM at a certain location if the species is specified. This converts volumetric stiffness into stiffness per 10 μm. It is then converted into unit in mN per meter (mN/m). For stiffness expressed in per unit CP length, the presented values are multiplied by 10 μm and then converted to unit of mN/m.

Magnitude of stiffness used in these cochlear models spread by more than 4 decades. In addition, as shown in Table S2, the base to apex ratio of stiffness also spreads widely, from 74 [[10](#_ENREF_10)] to 7300 [[8](#_ENREF_8),[11](#_ENREF_11)]. Note that the stiffness base to apex ratio measured in experiments [[12](#_ENREF_12),[13](#_ENREF_13)] are comparatively smaller than those used in cochlear models, for example, Naidu and Mountain’s [[13](#_ENREF_13)] measurement of the BM point stiffness after conversion is only ~120. To quantify the stiffness gradient across different species with different range of characteristic frequency, we suggest the stiffness gradient to be normalized by the specific frequency variation of a species.

| 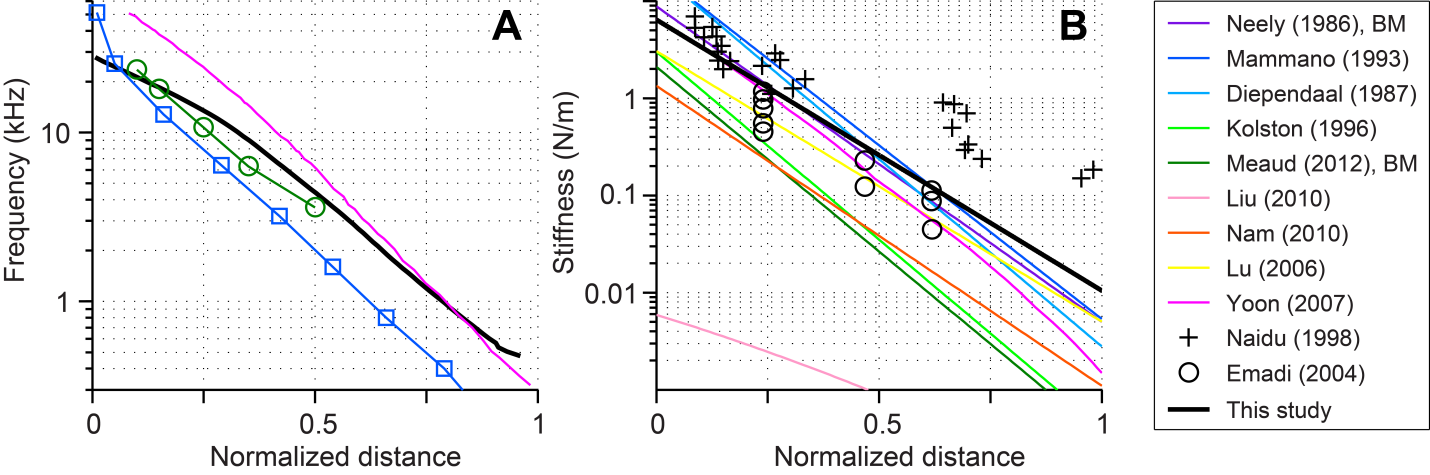 |
| --- |
| **Figure C. Comparison of frequency location relations and OCC stiffness per 10 μm section with other studies. (A)** Frequency-location relation active and **(B)** point stiffness measured and stiffness used in previous modeling studies were converted into stiffness per 10 μm section as described in the text. Frequency-location relations for four species are included: guinea pig [[9](#_ENREF_9),[14](#_ENREF_14),[15](#_ENREF_15)], chinchilla [[16](#_ENREF_16)], gerbil [[17](#_ENREF_17)] and cat [[18](#_ENREF_18)]. |

| 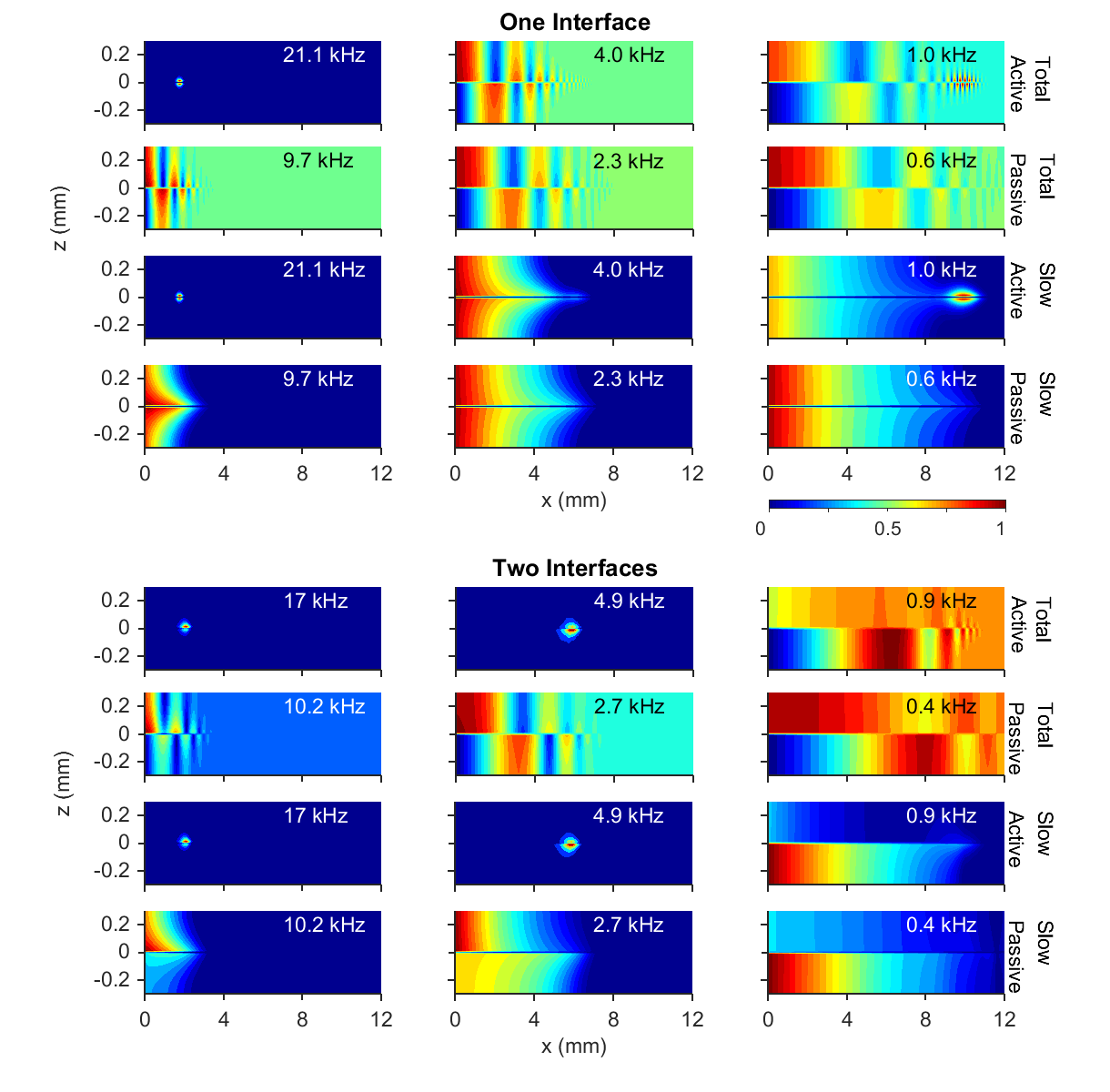 |
| --- |
| **Figure D. Cochlear fluid pressure: One and two fluid-interacting surfaces.** The contour plots represent the fluid pressure amplitude relative to the pressure amplitude at oval window under two conditions (active and passive), when the BM responses peak at x = 2, 6, and 10 mm. Total pressure (two upper row panels) and slow component of the fluid pressure (two lower row panels) are plotted. The slow component of fluid pressure was obtained by subtracting the pressure at the apical opening (the fast component). The pressure gain is defined as $p_{Total}=\left\vert p \right\vert/\left\vert p_{OW} \right\vert$, and $p_{Slow}=\left( \left\vert p \right\vert-\left\vert p_{HT} \right\vert\right)/\left\vert p_{OW} \right\vert$ where $p_{HT}$ and $p_{OW}$ are fluid pressures at the helicotrema and the oval window, respectively. The peak pressure gains are: [10000, 1, 1.5; 1, 1, 1; 1,0.5, 0.8; 0.5, 0.50, 0.5, 0.5] for the one interface case, and [200, 30, 2.6; 1, 1,1; 200, 30, 2; 0.8, 0.65, 0.7] for the two interfaces case, where the column and rows were separated by commas and semi-colons, respectively. |

# References

1. Prodanovic S, Gracewski SM, Nam J-H (2015) Power dissipation in the sub-tectorial space of the mammalian cochlea modulated by inner hair cell stereocilia. Biophys J.

2. Beurg M, Nam JH, Crawford A, Fettiplace R (2008) The actions of calcium on hair bundle mechanics in mammalian cochlear hair cells. Biophys J 94: 2639-2653.

3. Nam JH, Fettiplace R (2008) Theoretical conditions for high-frequency hair bundle oscillations in auditory hair cells. Biophys J 95: 4948-4962.

4. Beurg M, Nam JH, Chen Q, Fettiplace R (2010) Calcium balance and mechanotransduction in rat cochlear hair cells. J Neurophysiol 104: 18-34.

5. Beurg M, Fettiplace R, Nam JH, Ricci AJ (2009) Localization of inner hair cell mechanotransducer channels using high-speed calcium imaging. Nat Neurosci 12: 553-558.

6. Meaud J, Grosh K (2014) Effect of the Attachment of the Tectorial Membrane on Cochlear Micromechanics and Two-Tone Suppression. Biophysical Journal 106: 1398-1405.

7. Gavara N, Chadwick R (2009) Collagen-based mechanical anisotropy of the tectorial membrane: implications for inter-row coupling of outer hair cell bundles. PloS one 4.

8. Kolston P, Ashmore J (1996) Finite element micromechanical modeling of the cochlea in three dimensions. J Acoust Soc Am.

9. Mammano F, Nobili R (1993) Biophysics of the cochlea: linear approximation. J Acoust Soc Am 93: 3320-3332.

10. Liu YW, Neely ST (2010) Distortion product emissions from a cochlear model with nonlinear mechanoelectrical transduction in outer hair cells. J Acoust Soc Am 127: 2420-2432.

11. Diependaal R, Duifhuis H, Hoogstraten H, Viergever M (1987) Numerical methods for solving one-dimensional cochlear models in the time domain. J Acoust Soc Am 82: 1655-1666.

12. Emadi G, Richter CP, Dallos P (2004) Stiffness of the gerbil basilar membrane: radial and longitudinal variations. J Neurophysiol 91: 474-488.

13. Naidu RC, Mountain DC (1998) Measurements of the stiffness map challenge a basic tenet of cochlear theories. Hear Res 124: 124-131.

14. Meaud J, Grosh K (2012) Response to a pure tone in a nonlinear mechanical-electrical-acoustical model of the cochlea. Biophysical journal 102: 1237-1246.

15. Ramamoorthy S, Deo NV, Grosh K (2007) A mechano-electro-acoustical model for the cochlea: response to acoustic stimuli. J Acoust Soc Am 121: 2758-2773.

16. Lim KM, Steele CR (2002) A three-dimensional nonlinear active cochlear model analyzed by the WKB-numeric method. Hear Res 170: 190-205.

17. Yoon Y-J, Puria S, Steele C (2007) Intracochlear pressure and derived quantities from a three-dimensional model. The Journal of the Acoustical Society of America 122: 952-966.

18. Neely ST, Kim DO (1986) A model for active elements in cochlear biomechanics. J Acoust Soc Am 79: 1472-1480.
